# TIAM1 Antagonizes TAZ/YAP Both in the Destruction Complex in the Cytoplasm and in the Nucleus to Inhibit Invasion of Intestinal Epithelial Cells

**DOI:** 10.1016/j.ccell.2017.03.007

**Published:** 2017-05-08

**Authors:** Zoi Diamantopoulou, Gavin White, Muhammad Z.H. Fadlullah, Marcel Dreger, Karen Pickering, Joe Maltas, Garry Ashton, Ruth MacLeod, George S. Baillie, Valerie Kouskoff, Georges Lacaud, Graeme I. Murray, Owen J. Sansom, Adam F.L. Hurlstone, Angeliki Malliri

**Affiliations:** 1 Cell Signalling Group, Cancer Research UK Manchester Institute, The University of Manchester, Manchester M20 4BX, UK; 2 Stem Cell Haematopoiesis Group, Cancer Research UK Manchester Institute, The University of Manchester, Manchester M20 4BX, UK; 3 Stem Cell Biology Group, Cancer Research UK Manchester Institute, The University of Manchester, Manchester M20 4BX, UK; 4 School of Medical Sciences, Faculty of Biology, Medicine and Health, The University of Manchester, Manchester M13 9PT, UK; 5 Colorectal Cancer and Wnt Signalling, Cancer Research UK Beatson Institute, Glasgow G61 1BD, UK; 6 Histology, Cancer Research UK Manchester Institute, The University of Manchester, Manchester M20 4BX, UK; 7 Institute of Cardiovascular and Medical Science, College of Medical Veterinary and Life Sciences, University of Glasgow, Glasgow G12 8QQ, UK; 8 Pathology, School of Medicine, Medical Sciences and Nutrition, University of Aberdeen, Aberdeen AB25 2ZD, UK

**Keywords:** TIAM1, TAZ, YAP, WNT, intestinal tumorigenesis, colorectal cancer, cell migration, invasion, malignant progression

## Abstract

Aberrant WNT signaling drives colorectal cancer (CRC). Here, we identify TIAM1 as a critical antagonist of CRC progression through inhibiting TAZ and YAP, effectors of WNT signaling. We demonstrate that TIAM1 shuttles between the cytoplasm and nucleus antagonizing TAZ/YAP by distinct mechanisms in the two compartments. In the cytoplasm, TIAM1 localizes to the destruction complex and promotes TAZ degradation by enhancing its interaction with βTrCP. Nuclear TIAM1 suppresses TAZ/YAP interaction with TEADs, inhibiting expression of TAZ/YAP target genes implicated in epithelial-mesenchymal transition, cell migration, and invasion, and consequently suppresses CRC cell migration and invasion. Importantly, high nuclear TIAM1 in clinical specimens associates with increased CRC patient survival. Together, our findings suggest that in CRC TIAM1 suppresses tumor progression by regulating YAP/TAZ activity.

## Highlights

• TIAM1 is part of the WNT-regulated destruction complex regulating TAZ/YAP stability
• WNT induces TIAM1 nuclear translocation where it reduces TAZ/YAP-TEAD interaction
• Nuclear TIAM1 blocks the TAZ/YAP genetic program inhibiting migration of CRC cells
• Nuclear TIAM1 predicts good prognosis in CRC

## Graphical Abstract

**Figure:**
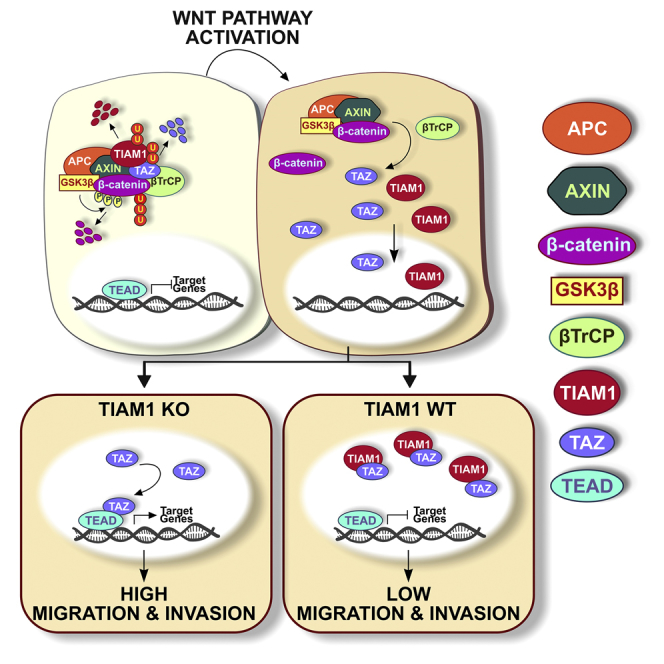

## Teaser

Diamantopoulou et al. identify TIAM1 as a critical antagonist of colorectal cancer progression. Cytoplasmic TIAM1 promotes TAZ degradation by enhancing its interaction with βTrCP whereas nuclear TIAM1 suppresses TAZ/YAP interaction with TEADs, inhibiting expression of TAZ/YAP target genes.

## Significance

**Colorectal carcinogenesis typically commences with constitutive WNT signaling leading to nuclear accumulation of transcriptional co-activators including TAZ and YAP. Thereafter**, **mutational and epigenetic events ensue inducing a genetic program that propels invasion and metastasis. We herein identify TIAM1 as a component of the cytoplasmic destruction complex regulating TAZ**/**YAP stability. We further show that when**, **as in initiated intestinal epithelial cells**, **the destruction complex is inactivated**, **TIAM1 along with TAZ**/**YAP accumulate and translocate from the cytoplasm to the nucleus. In the nucleus**, **however**, **TIAM1 continues to antagonize nuclear TAZ**/**YAP function despite constitutive WNT signaling, and thereby suppresses cell migration and invasion. In keeping with these findings**, **nuclear TIAM1 is downregulated in advanced colorectal cancer, and low nuclear TIAM1 predicts poor prognosis.**

## Introduction

Intestinal epithelial cells transform into malignant cells following mutations in various oncogenes and tumor suppressors that drive changes in programs of gene expression. Typically, the initiating event is inactivation of the tumor suppressor adenomatous polyposis coli (APC) or, less frequently, activation of β-catenin, resulting in constitutive activation of the canonical WNT pathway. At the core of this pathway is the regulation of β-catenin by a cytoplasmic destruction complex. In the absence of a WNT signal, the central scaffold protein AXIN within the destruction complex binds APC which efficiently delivers cytosolic β-catenin to be phosphorylated by CK1 and then GSK3β. Phosphorylated β-catenin is subsequently ubiquitylated by the E3 ligase βTrCP and degraded by the proteasome. Binding of WNT to its receptor causes inactivation of the destruction complex, stabilization, and nuclear translocation of β-catenin, and subsequent transcription by a β-catenin/TCF complex (Clevers, 2006).

The transcriptional co-activators TAZ and YAP (TAZ/YAP; originally identified as targets of the HIPPO pathway) have recently been established as additional regulatory components but also as effectors of canonical WNT signaling. In the cytoplasm, TAZ/YAP interact with β-catenin directly and restrict β-catenin nuclear translocation (Imajo et al., 2012). Cytoplasmic TAZ/YAP also interact with DVL, a regulator of the destruction complex, inhibiting DVL phosphorylation by CK1 and consequently inhibiting β-catenin activation (Varelas et al., 2010). A further study showed that in the absence of WNT, TAZ/YAP are essential for βTrCP recruitment to the destruction complex and β-catenin degradation (Azzolin et al., 2014). Thus, cytoplasmic TAZ/YAP emerge as inhibitors of WNT signaling and, therefore, potential suppressors of colorectal cancer (CRC). Inactivation of the destruction complex, either through WNT stimulation or, as occurs in CRC cells, inactivation of APC, results in not only β-catenin but also TAZ/YAP stabilization and the opportunity for TAZ/YAP to translocate to the nucleus (Azzolin et al., 2012, Azzolin et al., 2014). Significantly, in contrast to the ability of TAZ/YAP to downregulate β-catenin in the cytoplasm, nuclear YAP seems to cooperate with β-catenin to transactivate WNT target genes (Azzolin et al., 2012, Rosenbluh et al., 2012). TAZ/YAP stimulate target gene expression involved in cell proliferation, stem cell self-renewal, and tumorigenesis through binding TEAD family transcription factors in the nucleus (Zhang et al., 2009, Zhao et al., 2008). In keeping, TAZ/YAP are required for the overgrowth of intestinal crypts and formation of adenomas following APC inactivation (Azzolin et al., 2014, Gregorieff et al., 2015) and TAZ/YAP expression is associated with poor prognosis of CRC patients (Wang et al., 2013, Yuen et al., 2013). Thus, the interaction between TAZ/YAP and the WNT pathway is complex, highlighting our incomplete understanding of how the pathway is regulated. In the present study, we investigated the role of TIAM1, a guanine nucleotide exchange factor specific for RAC1, in colon cancer progression.

## Results

### Nuclear TIAM1 Expression in Human Tumors is a Prognostic Marker for CRC Progression

**Figure 1:**
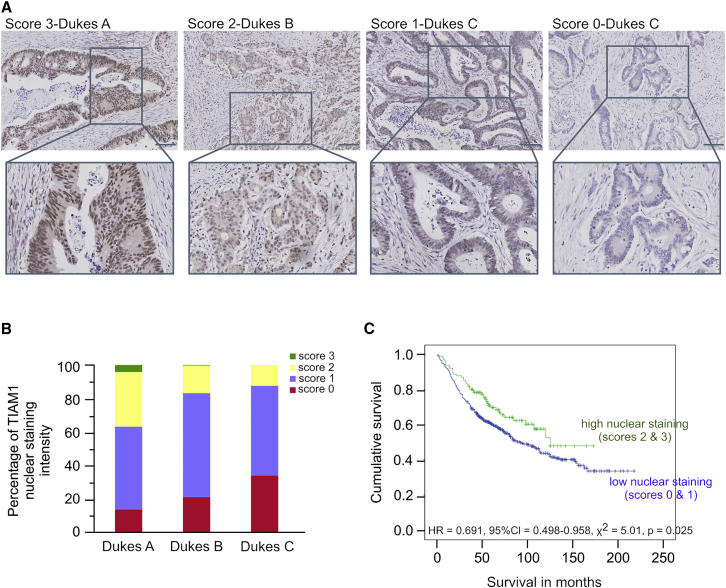
Abundance of Nuclear TIAM1 Impacts on CRC Progression (A) Immunohistochemical analysis of TIAM1 expression in a tissue microarray (TMA) of human colorectal cancers showing representative examples of strong (score 3), moderate (score 2), weak (score 1), and negative (score 0) staining. Scale bars, 100 μm. (B) Quantitation of TIAM1 nuclear staining intensity in CRC patients diagnosed with Dukes A, Dukes B, and Dukes C stage (χ^2^ = 54.165, p < 0.001). (C) Cumulative overall survival for patients with high (moderate/strong) and low (weak/negative) TIAM1-expressing tumors. HR, hazard ratio; CI, confidence interval. See also Figure S1 and Table S1.

Previously, using recombinant mouse models, we have shown that TIAM1 cooperates with WNT signaling during initiation of intestinal epithelial neoplasia but then appeared to antagonize tumor progression (Malliri et al., 2006). However, how TIAM1 influences intestinal tumor initiation and progression remained elusive. To further address the impact of TIAM1 on intestinal neoplasia and increase our understanding of its clinical significance, we probed a tissue microarray (TMA) comprising 650 samples, including Dukes stages A to C, from a well-characterized patient cohort (Brown et al., 2014) (Table S1), with a pre-validated TIAM1 antibody (Marei et al., 2016, Vaughan et al., 2015, Whalley et al., 2015). Intriguingly, we detected TIAM1 not only in the cytoplasm but also in cell nuclei (Figure 1A). Furthermore, nuclear TIAM1 staining intensity decreased with advancing Dukes stage (χ^2^ = 54.165, p < 0.001) (Figures 1A, 1B, and S1A). TIAM1 cytoplasmic staining also decreased with advancing Dukes stage (χ^2^ = 19.057, p = 0.001) (Figures 1A and S1B). Thus, TIAM1 expression is negatively associated with colon cancer progression, consistent with our previous finding that TIAM1 antagonized progression of intestinal tumors in *Apc*^*Min*/*+*^ mice (Malliri et al., 2006). To determine the association between TIAM1 expression and overall survival, we grouped nuclear TIAM1 staining into low (scores 0–1) or high (scores 2–3) categories and found that patients having CRC with high nuclear TIAM1 had significantly better survival than patients having CRC with low nuclear TIAM1 (Figures 1C and S1C). However, no difference was found in overall survival between patients having CRC expressing low or high cytoplasmic TIAM1 (Figure S1D). Thus, high levels of nuclear TIAM1 could serve as a good prognostic factor for CRC patients.

### TIAM1 Nucleocytoplasmic Shuttling is Regulated by a Functional Nuclear Localization Signal

**Figure 2:**
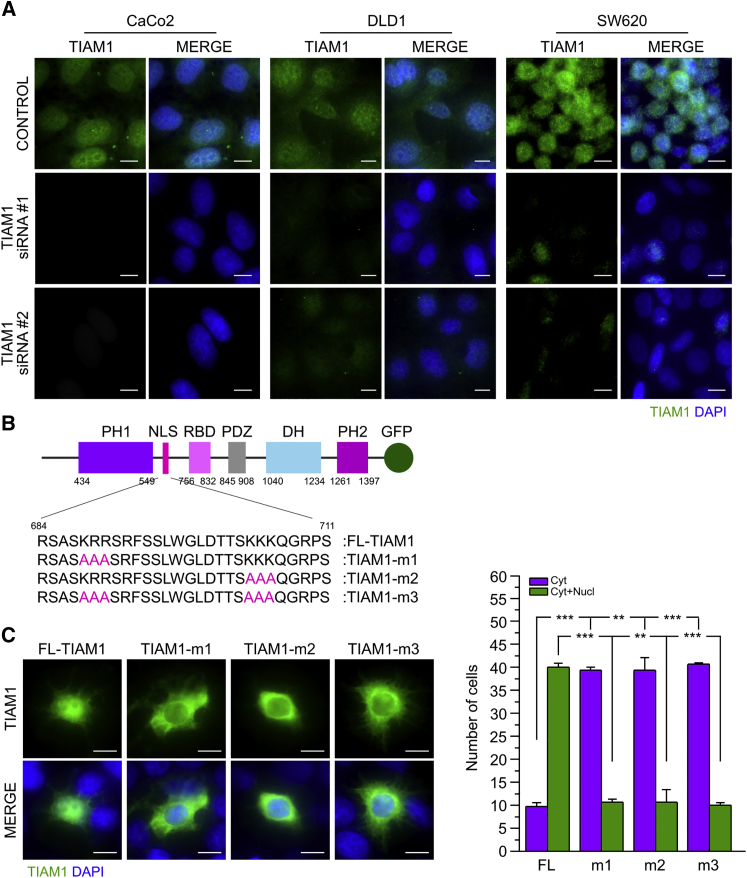
TIAM1 Nucleocytoplasmic Shuttling is Regulated by a Bipartite NLS (A) Representative confocal images of TIAM1 localization in three different CRC cell lines carrying APC mutations. TIAM1-depleted cells (TIAM1 siRNA) are used to demonstrate TIAM1 staining specificity. Scale bars, 10 μm. (B) Schematic representation of the position and sequence of the NLS of TIAM1 and the NLS mutants employed to study TIAM1 nucleocytoplasmic shuttling. (C) Representative confocal images of DLD1 cells transiently transfected with GFP-tagged FL-TIAM1 and the three different TIAM1-NLS mutants. Scale bars, 10 μm. Graph shows quantitation from three independent experiments (n = 50 cells per experiment). Data are presented as mean ± SEM, unpaired t test: ^∗∗^p < 0.01, ^∗∗∗^p < 0.001. See also Figure S2.

To validate TIAM1 nuclear localization, we analyzed its expression in CaCo2, DLD1, and SW620 CRC cell lines. TIAM1 was detected predominantly in nuclei from all three cell lines (Figure 2A). Furthermore, depletion of TIAM1 using two different small interfering RNAs (siRNAs) reported previously (Vaughan et al., 2015, Whalley et al., 2015) dramatically reduced the nuclear signal in all three lines (Figure 2A), verifying the specificity of the staining. Specificity of the nuclear TIAM1 staining was also verified in two independent clones of SW480 cells with TIAM1 ablated using CRISPR (Figures S2A–S2C).

We next addressed the mechanism of TIAM1 recruitment to the nucleus. Nuclear entry of proteins as large as TIAM1 that are unable to diffuse passively through nuclear pores is selective, dependent on the presence of a nuclear localization signal (NLS) (Freitas and Cunha, 2009). We therefore examined whether TIAM1 has a functional NLS. In silico analysis revealed two putative bipartite NLSs, but no monopartite NLS (Figure S2D). Between the two putative NLSs, the second had a high confidence score of 9.4 (Figure S2D), and aligning peptide sequences from different species revealed that NLS2 was more highly conserved (Figure S2E), implying an important regulatory function.

To investigate the functionality of the putative NLSs, we generated GFP-tagged TIAM1 constructs with the predicted NLSs deleted. The TIAM1ΔNLS1 construct contained a deletion in amino acids 269–298 and the TIAM1ΔNLS2 construct in amino acids 684–710. The constructs were transiently overexpressed in the DLD1 cell line and the number of cells with nuclear or non-nuclear localization defined by confocal microscopy. As expected, exogenously expressed full-length TIAM1 (FL-TIAM1-GFP) was detected both in the nucleus and the cytoplasm (Figure S2F), as was TIAM1-ΔNLS1 (data not shown); however, we observed significantly less TIAM1-ΔNLS2 in nuclei (Figure S2F). To further interrogate the role of NLS2 in controlling nuclear localization of TIAM1, we generated three different GFP-tagged TIAM1 mutants in which the clusters of basic residues ^688^KRR^690^ and ^704^KKK^706^ were mutated to AAA (Figure 2B). Cells expressing any of the three mutations in NLS2 displayed a significant decrease in nuclear TIAM1 (Figure 2C). Our results indicate, therefore, that TIAM1 shuttles between the cytoplasm and the nucleus mediated by at least one functional NLS.

### TIAM1 Antagonizes Transcription of TAZ/YAP Target Genes

**Figure 3:**
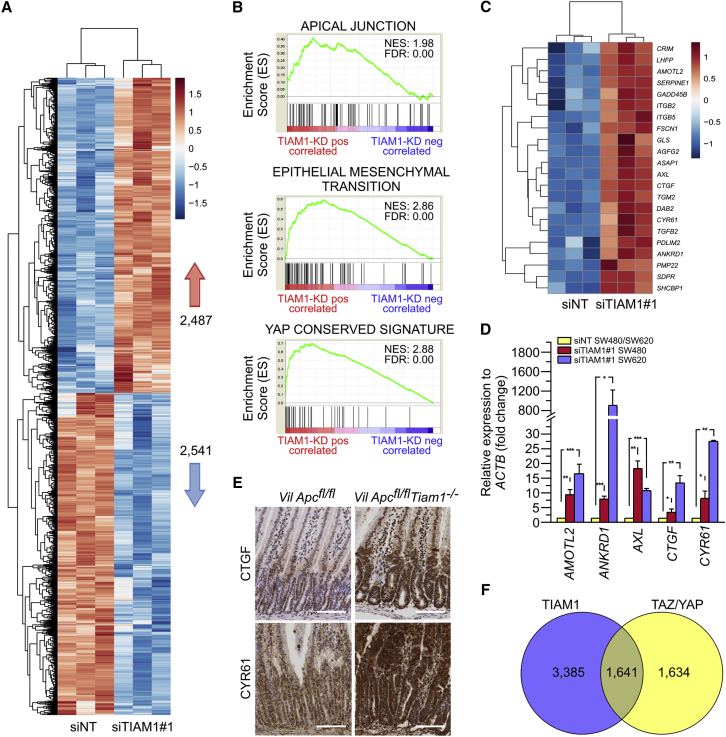
TIAM1 Suppresses a TAZ/YAP Transcriptional Program (A) Heatmap depiction of differentially expressed genes between siNT and siTIAM1#1 transfected SW620 cells from three independent experiments. A total of 2,487 genes (in red) are positively correlated with TIAM1 depletion and 2,541 genes (in blue) are negatively correlated. (B) GSEA with a significant enrichment score showing biological processes and signaling pathways that are positively correlated with TIAM1 knockdown in SW620 cells. FDR, false discovery rate; NES, normalized enrichment score. (C) Heatmap depiction of all differentially expressed genes between siNT and siTIAM1#1 transfected SW620 cells that contribute to the YAP conserved signature. (D) qPCR for TAZ/YAP target genes *AMOTL2*, *ANKRD1*, *AXL*, *CTGF*, and *CYR61* normalized to *ACTB* expression in SW620 and SW480 cells transfected with TIAM1#1 siRNA. Data are relative to negative control siRNA (siNT; set as 1) and are presented as mean ± SEM (unpaired t test: ^∗^p < 0.05, ^∗∗^p < 0.01, ^∗∗∗^p < 0.001). (E) Representative images of immunohistochemistry for CTGF and CYR61 in *Vil-Cre-ER*^*T2*^*Apc*^*fl*/*fl*^ and *Vil-Cre-ER*^*T2*^*Apc*^*fl*/*fl*^*Tiam1*^*−*/*−*^ intestines of tamoxifen-treated mice. Scale bars, 100 μm. (F) Venn diagram showing overlap of TIAM1 and TAZ/YAP target genes as determined by RNA-seq analysis (differentially expressed genes between siNT and siTAZ/YAP transfected SW620 cells were determined from three independent experiments). See also Figure S3; Tables S2 and S3.

The nuclear localization of TIAM1 suggested a possible role in regulating gene expression, whose interrogation might allow us to elucidate the contribution of TIAM1 to CRC development. By performing RNA sequencing (RNA-seq) on SW620 cells transfected with either siTIAM1#1 or a negative control siRNA (siNT) (Figure S3A), we identified TIAM1 differentially expressed genes (Table S2). The expression profiles of siNT and siTIAM1#1 samples were clearly separated based on principal component analysis (Figure S3B), and 5,027 genes were found to be differentially expressed (false discovery rate <0.05) between the two populations (Figure 3A). To surmise molecular pathways associated with TIAM1 regulated genes, we performed gene set enrichment analysis (GSEA) using gene sets for the oncogenic pathways in the Molecular Signature Database (MSigDB) (Subramanian et al., 2005). GSEA revealed significant enrichment among TIAM1-regulated genes for apical polarity and epithelial-mesenchymal transition (EMT) signatures (Figure 3B), cellular processes well known to be regulated by TIAM1 (Mack et al., 2012, Vaughan et al., 2015, Woodcock et al., 2009). Interestingly, we also found that TIAM1-regulated genes overlapped significantly with the YAP-associated molecular signature (Cordenonsi et al., 2011) (Figure 3B). Indeed, numerous well-characterized TAZ/YAP target genes were found to be upregulated in the RNA-seq dataset upon knockdown of TIAM1 (Figure 3C). Furthermore, five target genes (*AMOTL2*, *ANKRD1*, *AXL*, *CTGF*, and *CYR61*) were validated to be upregulated by qRT-PCR in both SW620 and SW480 cells using two independent siRNA sequences targeting TIAM1 (Figures 3D, S3C, and S3D). These findings indicated that TIAM1 might be antagonizing induction of TAZ/YAP target genes.

Given the important role of TAZ/YAP in CRC, we decided to investigate further the interplay of TIAM1 with TAZ/YAP. First, we tested whether the effect of TIAM1 depletion on the expression of CTGF and CYR61 was also observed in vivo. As shown in Figure 3E, the levels of both CTGF and CYR61 were upregulated in the small intestines of *Vil-Cre-ER*^*T2*^ *Apc*^*fl*/*fl*^ *Tiam1*^*−*/*−*^ compared with *Vil-Cre-ER*^*T2*^ *Apc*^*fl*/*fl*^ mice treated with tamoxifen. Second, using RNA-seq we generated a list of TAZ/YAP differentially expressed genes from SW620 cells transfected with either specific siRNAs for TAZ/YAP or a negative control siRNA (Figure S3E and Table S3) and compared it with the siTIAM1#1 RNA-seq dataset. Interestingly, we found that 50% of TAZ/YAP-regulated genes were also TIAM1 dependent (Figure 3F).

### TIAM1 Regulates TAZ Stability and Nuclear Translocation

**Figure 4:**
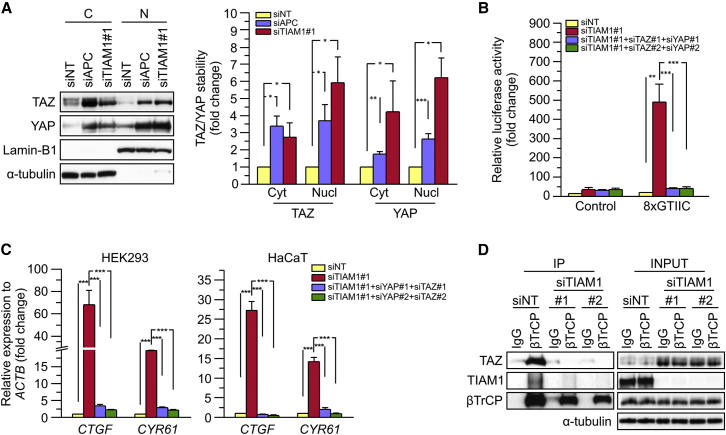
TIAM1 Regulates TAZ Stability and Nuclear Translocation (A) Western blot analyses of cytoplasmic (C) and nuclear (N) fractions from confluent HEK293 cells treated with APC or TIAM1#1 siRNAs. Graph shows mean TAZ and YAP levels normalized to cytoplasmic (α-tubulin) or nuclear (Lamin-B1) markers and then to negative control siRNA (siNT). (B) Luciferase assay for 8xGTIIC-Lux or control reporter indicating TAZ/YAP-dependent transcriptional activity in confluent HEK293 cells transfected with TIAM1#1 siRNA, either alone or with two different pairs of TAZ/YAP siRNAs as indicated. Data are normalized to a Renilla reporter and to negative control siRNA (siNT). (C) qPCR for TAZ/YAP target genes *CTGF* and *CYR61* normalized to *ACTB* expression in confluent HEK293 and HaCaT cells transfected with TIAM1#1 siRNA, either alone or with two different pairs of TAZ/YAP siRNAs as indicated. Data are normalized to negative control siRNA (siNT). (D) Western blot analyses of endogenous TAZ and TIAM1 co-immunoprecipitating (IP) with endogenous βTrCP from confluent HEK293 cells treated with TIAM1#1 or TIAM1#2 siRNA. Immunoglobulin G (IgG) was used as a control antibody for immunoprecipitation. Figure is representative of three independent experiments. Data shown in graphs are presented as mean ± SEM with siNT set as 1 (unpaired t test for three independent experiments: ^∗^p < 0.05, ^∗∗^p < 0.01, ^∗∗∗^p < 0.001). See also Figure S4.

The inverse association between TAZ/YAP and TIAM1-regulated genes prompted us to investigate the molecular mechanism(s) by which TIAM1 might antagonize TAZ/YAP. Depletion of TIAM1 in HEK293 cells (Figure S4A) using siTIAM1#1 resulted in a statistically significant increase of TAZ protein both in the cytoplasm and in the nuclei of HEK293 cells comparable with that induced by APC depletion (Figure 4A). A similar effect on TAZ was observed after depletion of TIAM1 by a second siRNA sequence (siTIAM1#2) (Figure S4B). Because TAZ mRNA levels were not significantly upregulated by TIAM1 depletion (Figure S4C), this suggested that TIAM1 depletion promoted the stabilization and nuclear translocation of TAZ. YAP stability, and nuclear translocation also appeared to increase upon TIAM1 and APC depletion (Figures 4A and S4B).

To further investigate the relationship between TIAM1 and TAZ/YAP, we tested the effect of TIAM1 depletion on the activity of 8xGTIIC-Lux, a synthetic luciferase reporter containing multimerized response elements of TEAD, the main DNA-binding cofactor of TAZ or YAP (Dupont et al., 2011). As shown in Figure 4B, TIAM1 depletion strongly induced the activity of 8xGTIIC-Lux, indicating that TIAM1 regulates TAZ/YAP activity. Depletion of endogenous TAZ alone partially rescued the effect of TIAM1 depletion (Figures S4D and S4E), while depletion of YAP or a combination of YAP and TAZ was even more effective at reversing the effect (Figures 4B, S4D, and S4E). Moreover, the expression of the endogenous TAZ/YAP target genes *CTGF* and *CYR61* was also shown to be upregulated by TIAM1 depletion in both HEK293 and HaCaT cells (Figures 4C and S4E–S4G). Again this effect could be reversed by depleting TAZ or YAP alone, and particularly through combined depletion of both (Figures 4C and S4E–S4G). Analysis of target gene expression and activity of a synthetic reporter thus confirmed the ability of TIAM1 to antagonize the function of both TAZ and YAP.

Finally, we decided to investigate the mechanism by which TIAM1 depletion induced the stability of TAZ. As previous studies have shown that the E3 ubiquitin ligase βTrCP interacts directly with both TAZ (Azzolin et al., 2012, Huang et al., 2012, Liu et al., 2010) and TIAM1 (Magliozzi et al., 2014, Zhu et al., 2014), we hypothesized that TIAM1 might regulate the interaction of TAZ with βTrCP. By carrying out immunoprecipitations for endogenous βTrCP from confluent control or TIAM1-depleted HEK293 cells, we found that the interaction of TAZ with βTrCP was decreased in cells in which TIAM1 expression was downregulated (Figure 4D). Taken together, these results indicate that TIAM1 is important for the interaction of TAZ with βTrCP through which it regulates the stability and cellular distribution of TAZ.

### TIAM1 is Regulated by the Canonical WNT Pathway

**Figure 5:**
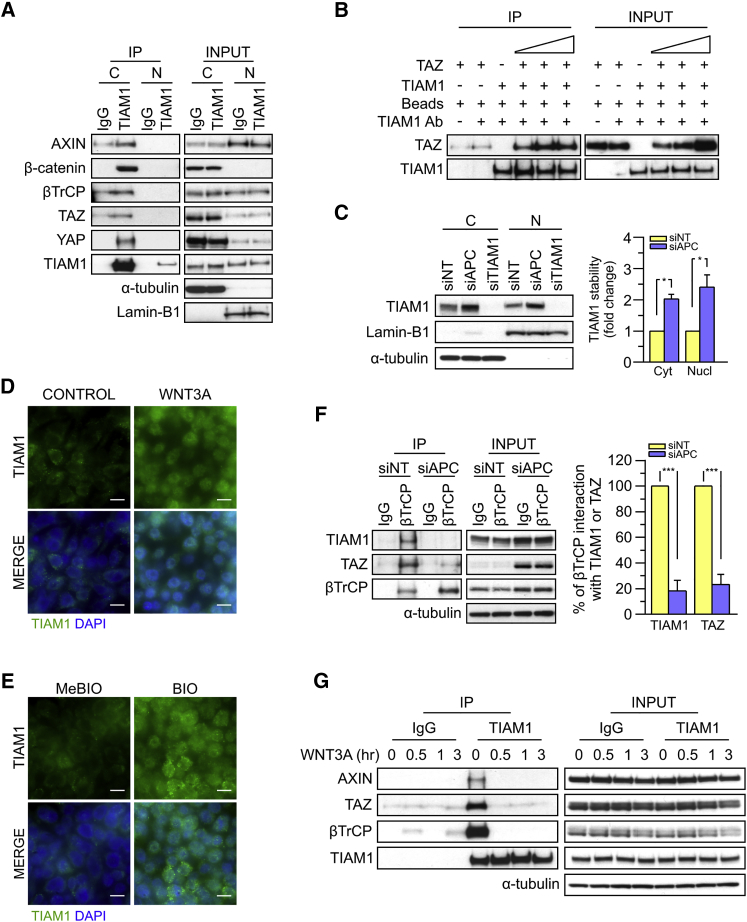
TIAM1 is Regulated by the Canonical WNT Pathway (A) Western blot analyses of endogenous proteins co-immunoprecipitating with endogenous TIAM1 from cytoplasmic (C) and nuclear (N) fractions of confluent HEK293 cells. IgG was used as a control antibody for immunoprecipitation. Figure is representative of three independent experiments. (B) Western blot analyses of increasing amounts of recombinant TAZ immunoprecipitated with recombinant TIAM1. Beads alone, no recombinant TIAM1, or no recombinant TAZ were used as negative controls. Figure is representative of three independent experiments. (C) Western blot analyses of cytoplasmic (C) and nuclear (N) fractions from confluent HEK293 cells transfected with APC siRNA. Lysates from TIAM1-depleted cells (TIAM1#1 siRNA) were used as specificity control for the TIAM1 antibody. Graph shows mean TIAM1 levels normalized to cytoplasmic (α-tubulin) or nuclear (Lamin-B1) markers and then to negative control siRNA (siNT). (D and E) Representative confocal images of TIAM1 localization in confluent RKO cells treated with control or WNT3A-conditioned medium (D) or control (MeBIO) or GSK3a inhibitor (BIO) (E). Images are representative of three independent experiments. Scale bars, 10 μm. (F) Western blot analyses of endogenous TIAM1 and TAZ co-immunoprecipitating (IP) with endogenous βTrCP from confluent HEK293 cells treated with APC siRNA. IgG was used as a control antibody for immunoprecipitation. Graph shows average βTrCP/TIAM1 or βTrCP/TAZ interaction from three independent replicates. (G) Western blot analyses of endogenous proteins co-immunoprecipitating with endogenous TIAM1 from confluent HEK293 cells treated with WNT3A conditioned medium for different time points as indicated. IgG was used as a control antibody for immunoprecipitations. Figure is representative of three independent experiments. Data shown in graphs are presented as mean ± SEM with siNT set as 1 or 100% (unpaired t test for three independent experiments: ^∗^p < 0.05, ^∗∗∗^p < 0.001). See also Figure S5.

As stated above, previous studies have shown that a major factor regulating the stability and nuclear translocation of TAZ is the destruction complex (Azzolin et al., 2012, Azzolin et al., 2014), and our aforementioned findings suggested that TIAM1 is also implicated in this mechanism of TAZ regulation. This prompted us to investigate whether TIAM1 was a component of the WNT-regulated destruction complex. By immunoprecipitating endogenous TIAM1 from cytosolic and nuclear fractions of confluent HEK293 cells, which carry a functional destruction complex, we found that TIAM1 interacted with AXIN, β-catenin, and (as shown previously by Magliozzi et al., 2014, Zhu et al., 2014, and above) βTrCP, which are established elements of the destruction complex as well as TAZ and YAP (Figure 5A). Even though low levels of TIAM1 were detected in the nucleus, TIAM1 interaction with AXIN, β-catenin, βTrCP, TAZ, and YAP was observed only in the cytoplasm of HEK293 cells. The interaction of TIAM1 with TAZ was confirmed in vitro using recombinant proteins and was inferred to be direct (Figure 5B).

Since the main function of the destruction complex is regulating protein stability through degradation, we wondered whether its inactivation would affect TIAM1 stability. The first approach we used for inactivation was downregulation of APC expression by a siRNA (Figure S5A). APC depletion resulted in a statistically significant increase in TIAM1 levels in the cytoplasm of HEK293 cells (Figure 5C). Since TIAM1 mRNA levels were not increased by APC depletion (Figure S5B), this suggested that APC depletion promoted the stabilization of TIAM1 protein. Interestingly, we also observed a significant increase in the nuclear levels of TIAM1 (Figure 5C). The stabilization and nuclear translocation of TIAM1 following APC depletion was also confirmed by immunofluorescence microscopy (Figure S5C) and is consistent with the nuclear accumulation of TIAM1 we observe in colorectal tumors (Figure 1A) and CRC cell lines with defective APC (Figure 2A). As a second approach to inactivate the destruction complex, we used conditioned medium from WNT3A expressing cells. Similar to APC depletion, treatment of confluent RKO cells with WNT3A-conditioned medium induced the stabilization and translocation of TIAM1 from the cytoplasm into the nucleus (Figure 5D). Finally, activation of the WNT pathway by BIO, a small molecule that inhibits GSK3β activity (Meijer et al., 2003), also induced the stabilization and nuclear accumulation of TIAM1 in RKO cells (Figure 5E). In summary, these results indicate that the WNT pathway regulates the stability and nuclear translocation of TIAM1 through the destruction complex, as described previously for TAZ (Azzolin et al., 2012, Azzolin et al., 2014).

We next addressed how the destruction complex regulates TIAM1 stability. It is known that βTrCP is responsible for the degradation of β-catenin (Hart et al., 1999, Kitagawa et al., 1999, Marikawa and Elinson, 1998) and TAZ (Azzolin et al., 2012, Huang et al., 2012, Liu et al., 2010). Previous studies have shown that TIAM1 also interacts with βTrCP, resulting in its degradation (Magliozzi et al., 2014, Zhu et al., 2014). This prompted us to investigate whether the increased stabilization of TIAM1 observed upon WNT3A treatment or APC depletion is due to its decreased interaction with βTrCP. By carrying out immunoprecipitations for endogenous βTrCP from confluent control or APC-depleted HEK293 cells, we found that the interaction of TIAM1 with βTrCP was almost abolished in cells in which APC was depleted (Figure 5F). A dramatic decrease in the interaction between TAZ and βTrCP was also observed under these conditions (Figure 5F). Furthermore, following activation of the WNT pathway by WNT3A treatment, TIAM1 interaction with proteins of the destruction complex including AXIN and βTrCP, as well as TAZ, was abolished (detected from 0.5 hr; Figure 5G). Taken together, these data imply that activation of the canonical WNT pathway (inactivation of the destruction complex) induces TIAM1 stability and nuclear translocation by regulating the interaction between TIAM1 and βTrCP.

### Nuclear TIAM1 Antagonizes TAZ Transcriptional Activity

**Figure 6:**
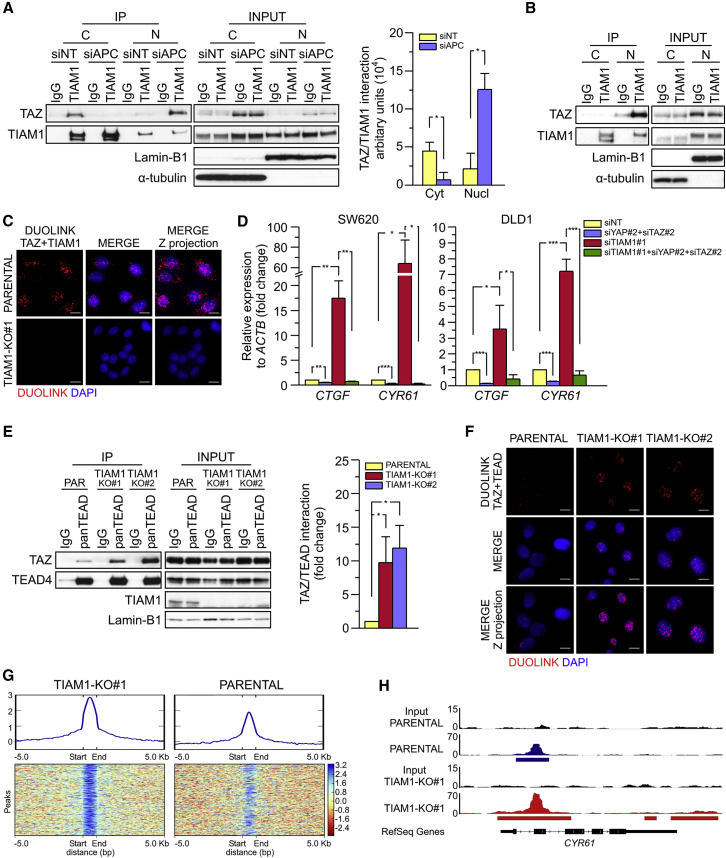
Nuclear TIAM1 Antagonizes TAZ Transcriptional Activity (A) Western blot analyses of endogenous TAZ co-immunoprecipitating (IP) with endogenous TIAM1 from cytoplasmic (C) and nuclear (N) fractions of confluent HEK293 cells treated with APC or a negative control (siNT) siRNA. IgG was used as a control antibody for immunoprecipitation. Graph shows mean levels of immunoprecipitated TAZ normalized to levels of immunoprecipitated cytoplasmic or nuclear TIAM1. (B) Western blot analyses of endogenous TAZ co-immunoprecipitating with endogenous TIAM1 from cytoplasmic (C) and nuclear (N) fractions of DLD1 cells. IgG was used as a control antibody for immunoprecipitation. Figure is representative of three independent experiments. (C) Representative confocal images from three independent experiments of TAZ/TIAM1 interaction in parental SW480 cells detected by Duolink assay. Staining in one CRISPR-mediated TIAM1 knockout clone (TIAM1-KO#1) was used as a control for antibody specificity. Scale bars, 10 μm. (D) qPCR for the TAZ/YAP target genes *CTGF* and *CYR61* normalized to *ACTB* expression in SW620 and DLD1 cells transfected with TIAM1#1 siRNA alone, TAZ/YAP siRNAs alone, or a combination of TIAM1#1 siRNA and TAZ/YAP siRNAs as indicated. Data are normalized to negative control siRNA (siNT). (E) Western blot analyses of endogenous TAZ co-immunoprecipitating with endogenous TEAD from nuclear fractions of parental SW480 cells or the CRISPR-mediated TIAM1 knockout clones. IgG was used as a control antibody for immunoprecipitation. Graph shows mean levels of immunoprecipitated TAZ normalized to levels of immunoprecipitated TEAD. (F) Representative confocal images from three independent experiments of TAZ/TEAD interaction in parental SW480 cells or the CRISPR-mediated TIAM1 knockout clones detected by Duolink assay. Scale bars, 10 μm. (G) Heatmap depiction of TAZ recruitment to chromatin of parental or CRISPR-mediated TIAM1-knockout clone #1 (TIAM1-KO#1) SW480 cells. (H) Read density tracks of TAZ ChIP-seq enrichment in *CYR61* locus of parental or CRISPR-mediated TIAM1-KO#1 SW480 cells. Data shown in graphs are presented as mean ± SEM with siNT or parental set as 1 in (D) and (E), respectively (unpaired t test for three independent experiments: ^∗^p < 0.05, ^∗∗^p < 0.01, ^∗∗∗^p < 0.001). See also Figure S6.

The role of TIAM1 in promoting TAZ degradation by the destruction complex could offer one explanation for the stimulation of TAZ transcriptional activity following TIAM1 depletion (Figures 4B and 4C). However, our findings also indicated that TIAM1 antagonizes TAZ transcriptional activity in CRC cells with a constitutively defective destruction complex (Figure 3), pointing to an additional mechanism by which TIAM1 antagonizes TAZ. Since inactivation of the destruction complex induces nuclear translocation of both TIAM1 and TAZ, we hypothesized that the two proteins might also interact in the nucleus, potentially interfering with TAZ transcriptional activity. Immunoprecipitation of endogenous TIAM1 from control or APC-depleted HEK293 cells revealed that inactivation of the destruction complex indeed resulted in the appearance of a nuclear TIAM1/TAZ complex accompanied by reduction of this complex in the cytoplasm (Figure 6A). An interaction between TIAM1 and TAZ could also be detected in nuclear extracts from three different CRC cell lines (Figures 6B, S6A, and S6B), and was confirmed by Duolink proximity ligation assay (Figure 6C). Next, we tested the effect of TIAM1 knockdown on TAZ transcriptional activity in these CRC cells. Similar to HEK293 and HaCaT cells (Figures 4C and S4E–S4G), TIAM1 depletion in SW620 and DLD1 cells strongly induced the expression of *CTGF* and *CYR61*, which could be suppressed by depletion of endogenous YAP and TAZ (Figures 6D and S6C), indicating that TIAM1 inhibits the transcriptional activity of YAP and TAZ in CRC cells.

The antagonistic effect of nuclear TIAM1 on TAZ/YAP activity prompted us to examine whether nuclear TIAM1 might prevent the interaction of TAZ and YAP with TEADs. Immunoprecipitating endogenous TEADs from nuclear extracts of parental SW480 cells or the two CRISPR-mediated TIAM1 knockout SW480 clones revealed that TIAM1 ablation increased the interaction of TEADs with TAZ and YAP (Figures 6E and S6D). Duolink proximity ligation assays confirmed that the interaction between TAZ or YAP with TEADs was increased in the absence of TIAM1 (Figures 6F and S6E). The inhibitory effect of nuclear TIAM1 on TAZ activity was further demonstrated by chromatin immunoprecipitation sequencing (ChIP-seq) analysis. Parental SW480 cells or the TIAM1 knockout SW480 clone #1 cells were used to determine the recruitment of TAZ to chromatin in the presence or absence of TIAM1. We found that the number of TAZ peaks identified in the TIAM1 knockout cells (35,450 peaks) was increased compared with the parental cells (24,913 peaks) (GEO: GSE90492). As shown in Figure S6F, of the total peaks discovered by MACS peak caller, a subset of the peaks were exclusively found in either the TIAM1 knockout SW480 clone #1 cells (23,849 peaks) or parental cells (13,101 peaks). We then examined locations with overlapping TAZ peaks. In total there were 5,740 overlapping peaks, of which 2,082 had higher (>1.5 fold) TAZ recruitment to the chromatin in the TIAM1 knockout cells compared with parental cells (Figure 6G). In contrast, only 84 peaks were found to have a higher TAZ recruitment in the parental cells. Furthermore, consistent with the above findings that TIAM1 knockdown induces the interaction of TAZ with TEAD and induces the expression of *CYR61*, we found that deletion of TIAM1 increased the binding of TAZ to the promoter region of *CYR61* (Figure 6H). Taken together, our results indicate that nuclear TIAM1 functions as a negative regulator of the transcriptional activity of TAZ and YAP.

### The Effect of Nuclear TIAM1 on CRC Cell Proliferation, Migration, and In Vivo Invasion

Having demonstrated that TIAM1 antagonizes TAZ/YAP transcriptional activity, we analyzed the functional significance of their interaction. Previous studies have shown that both TIAM1 and TAZ/YAP regulate cell proliferation and migration (Minard et al., 2004, Malliri et al., 2006, Zanconato et al., 2016). First, we evaluated the potential effect of TIAM1 depletion on the proliferation of CRC cell lines and their dependence on TAZ/YAP. TIAM1, TAZ/YAP, or a combination of all three was depleted using siRNAs, and cell number was determined 6 days post transfection. As shown in Figure S7A, downregulation of either TAZ/YAP or TIAM1 decreased the proliferation of DLD1, SW480, and SW620 cells. However, the combined depletion of TIAM1 and TAZ/YAP on the proliferation of CRC cells was not additive, indicating that the effect of TIAM1 depletion on cell proliferation is TAZ/YAP independent.

To investigate specifically the role of nuclear TIAM1 on cell proliferation, we generated an NLS-TIAM1 construct by fusing full-length TIAM1 with the well-characterized NLS of SV40 large T antigen. We verified that the NLS-TIAM1 construct was exclusively nuclear compared with full-length TIAM1 (FL-TIAM1) (Figure S7B). Expression of NLS-TIAM1 (resistant to siTIAM1#1) at levels comparable with endogenous TIAM1 in DLD1 or SW620 cells had no effect on the proliferation of TIAM1-depleted cells (Figure S7C). However, FL-TIAM1 partially restored proliferation in TIAM1-depleted cells. Similarly, expression of FL-TIAM1, but not NLS-TIAM1, in SW480 CRISPR-mediated TIAM1 knockout cells restored proliferation to levels observed in parental cells (Figure S7D). Taken together, these results indicate that the effect of TIAM1 depletion on cell proliferation is TAZ/YAP independent and not related to its nuclear localization.

**Figure 7:**
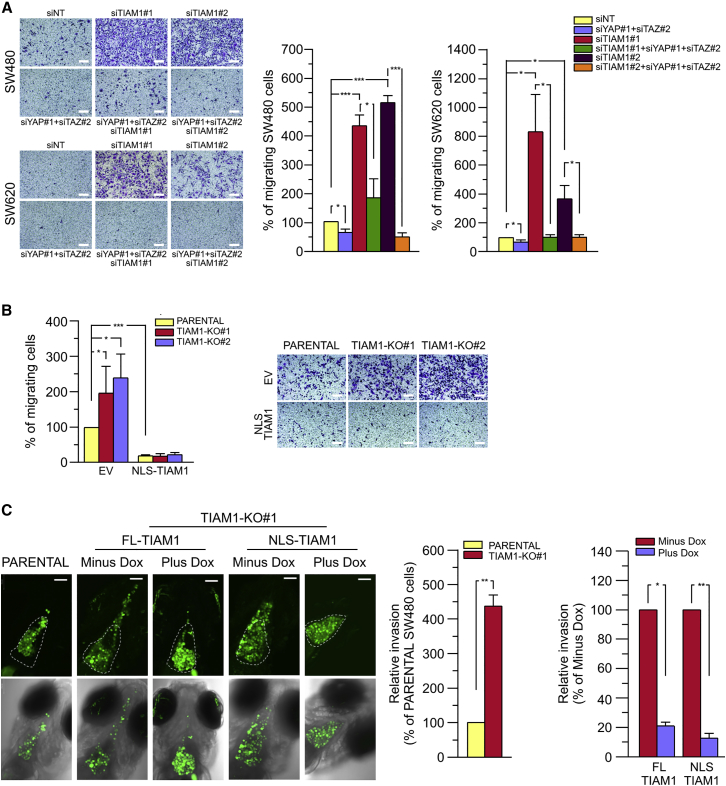
Effect of TIAM1 on Cell Migration and In Vivo Invasion (A) Representative images of SW480 cells or SW620 cells migrating through Transwell inserts toward serum for 24 hr. Cells were transfected with siRNA for TIAM1 or TAZ/YAP either alone or in combination, as indicated. Scale bars, 150 μm. Graphs show average number of migrating cells normalized to negative control siRNA (siNT) and are presented as mean ± SEM (with siNT set as 100) (unpaired t test: ^∗^p < 0.05, ^∗∗∗^p < 0.001). (B) Representative images of parental SW480 or the CRISPR-mediated TIAM1 knockout clones (TIAM1-KO #1 and # 2) migrating through Transwell inserts toward serum for 24 hr. Cells were transfected with NLS-TIAM1 or empty vector (EV) as control. Scale bars, 150 μm. Graph shows average number of migrating cells normalized to EV and are presented as mean ± SEM (EV transfected parental cells set as 100) (unpaired t test: ^∗^p < 0.05, ^∗∗∗^p < 0.001). (C) Representative images at 4 days post injection of parental green fluorescent SW480 and SW480-TIAM1-KO#1 cells, non-induced (Minus Dox), or inducibly expressing (Plus Dox) either FL-TIAM1 or NLS-TIAM1 engrafted into the pericardial cavity of zebrafish embryos. Upper row shows epifluorescence of injected cells. White dotted line indicates the pericardial cavity. Lower row shows merge with bright field. Scale bar, 100 μm. Graphs show quantitation of invasion. Mean values ± SEM from three independent experiments (Kruskal-Wallis one-way ANOVA and Dunn's post hoc test: ^∗^p < 0.05, ^∗∗^p < 0.01). See also Figure S7.

We next investigated the potential effect of TIAM1 depletion on the migration of SW480 and SW620 cells and its dependence on TAZ/YAP. TIAM1, TAZ/YAP, or a combination of all three was depleted using siRNAs, and cells were assayed for their migratory potential over 24 hr using the Boyden chamber assay. As shown in Figure 7A, TIAM1 depletion in both SW480 and SW620 cells induced a strong migratory response. Interestingly, this increase in migration was dependent on TAZ/YAP, as depletion of endogenous YAP and TAZ dramatically reduced the effect of TIAM1 depletion (Figure 7A). To verify that specifically nuclear TIAM1 suppresses cell migration, we analyzed the migratory capacity of SW480 cells overexpressing NLS-TIAM1. Notably, compared with parental SW480 cells, cells expressing NLS-TIAM1 migrated significantly less (Figure 7B). Furthermore, overexpression of NLS-TIAM1 abolished the increased migration observed in the two CRISPR-mediated TIAM1 knockout SW480 clones (Figure 7B).

To investigate the role of nuclear TIAM1 on migration in vivo, we turned to a zebrafish xenograft model. Fluorescently labeled parental SW480 cells or the aforementioned SW480 CRISPR-mediated TIAM1 knockout cells inducibly expressing either FL-TIAM1 or NLS-TIAM1 (Figure S7D) were injected into the pericardial cavity of 2-day-old zebrafish embryos. Ninety-six hours after injection, tumor cells were observed to have largely filled the approximately conical-shaped cavity (excluding the volume occupied by the heart) in all cases. Compared with parental SW480 cells, significantly more non-induced (minus doxycycline [Dox]) SW480 TIAM1 knockout cells disseminated away from the pericardial region (Figure 7C), whereas cells expressing (plus Dox) either FL-TIAM1 or equally NLS-TIAM1 showed a decreased ability to disseminate (Figure 7C), indicating the inhibitory role of nuclear TIAM1 on invasion. Taken together, these results suggest that nuclear TIAM1 suppresses TAZ/YAP-induced cell migration and in vivo invasion of CRC cells.

## Discussion

**Figure 8:**
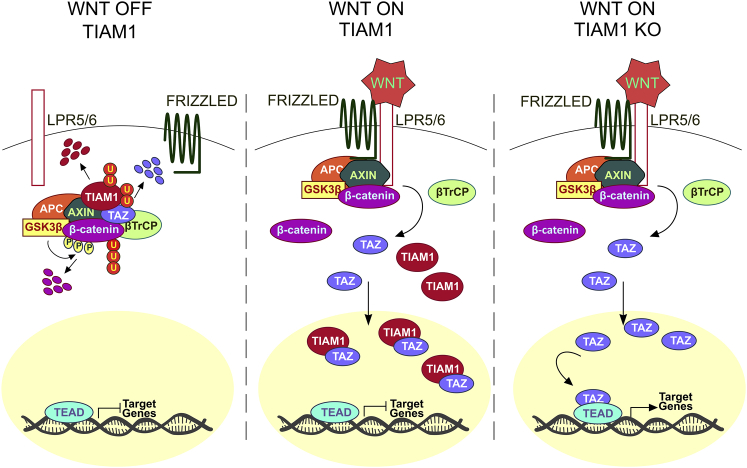
Model Depicting the Proposed Mechanism of TIAM1-Mediated Regulation of TAZ

Herein, we present evidence indicating that TIAM1 suppresses CRC cell migration and invasion by regulating TAZ/YAP transcriptional activity. Furthermore, we propose that this function of TIAM1 underpins our earlier observation that TIAM1 antagonizes malignant progression of intestinal neoplasia in the *Apc*^*Min*/*+*^ mouse model (Malliri et al., 2006). Interestingly, we found that the role of TIAM1 in regulating TAZ/YAP entails not only functions performed in the cytoplasm but also events in the nucleus, and propose the following model (Figure 8). (1) In the absence of WNT signaling, TIAM1 and TAZ are collectively recruited to the destruction complex for targeted ubiquitylation and degradation. (2) Upon WNT stimulation (or following initiating oncogenic events that inactivate the destruction complex), both TIAM1 and TAZ are released from the destruction complex and stabilized, and translocate to the nucleus. However, TAZ transcriptional activity is still greatly suppressed by the nuclear pool of TIAM1, impairing the interaction of TAZ with TEADs. (3) Upon WNT stimulation/inactivation of the destruction complex and in the absence of TIAM1, TAZ present in the nucleus transactivates the TEAD transcriptional program, which in the context of neoplasia would promote a more malignant phenotype.

One of the main findings of this study is that TIAM1 associates with the destruction complex. Inactivation of the destruction complex by various means was shown to promote TIAM1 accumulation and, concomitantly, its nuclear translocation. We further demonstrated that TIAM1 modulates the interaction between TAZ and βTrCP, influencing TAZ stability. As TIAM1 and βTrCP interact (Magliozzi et al., 2014, Zhu et al., 2014) as do TIAM1 and TAZ, it is probable that TIAM1 functions as a physical scaffold bringing TAZ and βTrCP into proximity. Whether TIAM1 directly interacts with AXIN, the main structural component of the destruction complex, or APC is presently unknown. However, the ability to immunoprecipitate a complex containing all these components suggests that the interactions are relatively stable.

The destruction complex is a key checkpoint for regulating canonical WNT signaling. Inactivation of the destruction complex liberates two different effectors: the well-documented β-catenin (Clevers, 2006) and recently implicated TAZ/YAP (Azzolin et al., 2012, Azzolin et al., 2014). Indeed, both transcriptional co-activators appear to be required for expression of WNT target genes in intestinal epithelial progenitor cells and CRC cells (Azzolin et al., 2012, Fevr et al., 2007). However, the identities of the transcription factor complexes that regulate subsets of WNT-induced target genes responsible for distinct WNT-mediated biological responses (such as stem cell maintenance, transit amplification, EMT, or terminal differentiation) have not been fully delineated. Previous studies have shown that TAZ is not required for β-catenin/TCF-dependent transcription (Azzolin et al., 2012, Imajo et al., 2015) and conversely that TAZ/YAP transactivation is independent of β-catenin/TCF (Imajo et al., 2015). Nonetheless, a complex between β-catenin and TAZ/YAP that includes the transcription factor TBX5 and is critical for β-catenin-driven transformation has been described (Rosenbluh et al., 2012). These studies indicate the complexity of the system and suggest that additional studies are required to fully uncover the transcriptional molecular mechanisms that regulate CRC initiation and progression.

Our study sheds light on TAZ/YAP function in CRC progression. Our results showed that nuclear TIAM1 plays a critical role in regulating the TAZ/YAP transcriptional program that is responsible for cell migration, with TIAM1 suppressing this program even when the WNT pathway is activated. Interestingly, the inhibitory nuclear function of TIAM1 on TAZ/YAP transcriptional activity is not associated with cell proliferation. Since one of the main molecular signatures that were enriched in the TIAM1-depleted cells was associated with EMT, we speculate that TIAM1 may be associated with suppressing cancer stem cell identity, since cancer stem cells are characterized by a mesenchymal cell gene expression program (Oskarsson et al., 2014). Furthermore, TAZ and YAP have been implicated in the expansion of stem cells in the regenerating gut but also in adenomatous polyps (Cai et al., 2010, Gregorieff et al., 2015). Our previous work revealed a gradient of TIAM1 expression highest toward the bottom of intestinal crypts, overlapping with the stem cell and transit amplification (TA) compartments (Malliri et al., 2006). In accordance with TIAM1 playing a role in suppressing stemness in the TA compartment through inhibiting TAZ/YAP, in this study we found that the TIAM1 knockout-mediated induction of the TAZ target genes *CTGF* and *CYR61* was predominantly observed in the expanded crypts of APC-deficient intestines.

Different studies have shown that APC loss alone is insufficient to promote intestinal tumor progression (Anderson et al., 2002, Blaker et al., 2003); additional mutations in other oncogenes such as *KRAS* and tumor-suppressor genes such as *TP53* are required (Fujii et al., 2016). Our results suggest that TIAM1 is an additional protein that needs to be inactivated during CRC progression. In support of this statement, our clinical data revealed that TIAM1 expression levels decline with disease progression and that patients having CRC with high levels of nuclear TIAM1 survive longer. Based on the outcome of cancer genome resequencing, TIAM1 downregulation is not typically achieved through mutation. Downregulation could be achieved at the transcriptional level or post-translationally. Previous work has shown that oncogenic RAS expression suppresses TIAM1 transcription (Zondag et al., 2000). Furthermore, we and others have identified E3 ubiquitin ligases targeting TIAM1 for degradation (Genau et al., 2015, Magliozzi et al., 2014, Vaughan et al., 2015, Zhu et al., 2014), which may be deregulated in intestinal carcinoma. Potentially, factors resulting in exclusion of TIAM1 from the nucleus might also result in downregulation of this critical suppressor function of TIAM1, and we are currently investigating the mechanisms regulating TIAM1 nuclear import and export. Previous studies have indicated that YAP and TAZ expression is increased in CRC (especially high-grade CRC), and their expression may be used as a prognostic marker (Cai et al., 2010, Wang et al., 2013, Yuen et al., 2013). However, TAZ/YAP expression levels might not be a sufficient prognostic marker for CRC patients, as differences in nuclear levels of TIAM1 might differentially affect the transcriptional activity of TAZ/YAP. An index based on both TAZ/YAP expression and nuclear TIAM1 intensity could therefore provide clearer prognostication.

## STAR★Methods

### Key Resources Table

**Table:** 

REAGENT or RESOURCE	SOURCE	IDENTIFIER
**Antibodies**		

Rabbit anti-TIAM1 Antibody, Affinity Purified	Bethyl Laboratories, Inc.	Cat# A300-099A RRID:AB_2271617
Polyclonal Sheep Human/Mouse TIAM1 Antibody	R&D Systems	Cat# AF5038 RRID:AB_2303175
Purified Mouse anti-TAZ Clone M2-616 (RUO)	BD Biosciences	Cat# 560235 RRID:AB_1645338
Anti-WWTR1 antibody produced in rabbit	Sigma-Aldrich	Cat# HPA007415 RRID:AB_1080602
YAP (D8H1X) XP® Rabbit mAb	Cell Signaling Technology	Cat# 14074
Lamin B1 (D4Q4Z) Rabbit mAb	Cell Signaling Technology	Cat# 12586
β-TrCP (D13F10) Rabbit mAb	Cell Signaling Technology	Cat# 4394S RRID:AB_10545763
Anti-BTRC Antibody (1B1D2)	Invitrogen	Cat# 373400 RRID:AB_431439
Axin1 (C76H11) Rabbit mAb	Cell Signaling Technology	Cat# 2087 RRID:AB_2274550
β-Catenin (D10A8) XP® Rabbit mAb	Cell Signaling Technology	Cat# 8480 RRID:AB_11127855
Anti-TEAD4 antibody - ChIP Grade	Abcam	Cat# ab58310 RRID:AB_945789
Pan-TEAD (D3F7L) Rabbit mAb	Cell Signaling Technology	Cat# 13295
CTGF Antibody (L-20) goat polyclonal	Santa Cruz Biotechnology	Cat# sc-14939 RRID:AB_638805
Mouse anti-α-Tubulin Monoclonal antibody, Clone DM1A	Sigma-Aldrich	Cat# T9026 RRID:AB_477593
Rabbit Anti-CTGF antibody Polyclonal antibody	Abcam	Cat# ab6992 RRID:AB_305688
Anti-CCN1 (CYR61) antibody	Abcam	Cat# ab24448 RRID:AB_2088724

**Biological Samples**		

CRC TMA	Prof. Graeme I. Murray	Brown et al., 2014

**Chemicals**, **Peptides and Recombinant Proteins**		

Recombinant TAZ	Abnova	Cat# H0006901-P01
BIO	Tocris Bioscience	Cat# 3194
MeBIO	Tocris Bioscience	Cat# 3873
NUPAGE 3-8% TA GEL 1.0MM10W	ThermoFischer Scientific	Cat# EA0375BOX
GammaBind™ G Sepharose™	GE Healthcare Life Sciences	Cat# GE17-0885-01
Doxycycline	Sigma-Aldrich	Cat# D9891
Lipofectamine® RNAiMAX Transfection Reagent	ThermoFischer Scientific	Cat# 13778075
*Trans*IT®-LT1 Transfection Reagent	Mirus	Cat# MIR 2300
PfuUltra II Fusion HS DNA Polymerase	Agilent Technologies	Cat# #600672
Tamoxifen	Sigma-Aldrich	Cat# H7904
Polyvinylpyrrolidinone solution K60	Sigma-Aldrich	Cat# 81430
Ethyl 3-aminobenzoate methanesulfonate (MS-222)	Sigma-Aldrich	Cat# E10521

**Critical Commercial Assays**		

RNeasy Mini Kit	Qiagen, UK	Cat# 74104
Omniscript RT Kit	Qiagen, UK	Cat# 205111
QuikChange II XL Site-Directed Mutagenesis Kit	Agilent Technologies	Cat# 200522
Dual-Luciferase Reporter Assay System	Promega	Cat# E1910
SimpleChIP® Enzymatic Chromatin IP Kit (Magnetic Beads)	Cell Signaling Technology	Cat# 9003
MicroPlex Library Preparation Kit v2	Diagenode	Cat# C05010014
Duolink® In Situ Red Starter Kit Mouse/Rabbit	Sigma-Aldrich	Cat# DUO92101
Duolink® In Situ Red Starter Kit Goat/Rabbit	Sigma-Aldrich	Cat# DUO92105

**Deposited Data**		

RNA-seq and ChIP-seq data	This study	GEO: GSE90492

**Experimental Models**: **Cell Lines**		

CaCo2	Gift from Prof Hans Clevers	N/A
DLD1	Gift from Prof Hans Clevers	Authenticated by MBCF of CRUK MI
SW480	Gift from Prof Hans Clevers	Authenticated by MBCF of CRUK MI
SW620	Gift from Prof Hans Clevers	Authenticated by MBCF of CRUK MI
HEK293	ECACC (operated by Public Health England)	Cat# 85120602
HaCaT	Gift from Dr Vania Braga	N/A
RKO	Prof Owen Sansom lab	N/A
L-WNT3a cells	Prof Owen Sansom lab	N/A
L-Control cells	Prof Owen Sansom lab	N/A
SW480-TIAM1-KO#1	This study	N/A
SW480-TIAM1-KO#2	This study	N/A
SW480-TIAM1-KO#1-FL-TIAM1	This study	N/A
SW480-TIAM1-KO#1-NLS-TIAM1	This study	N/A
DLD1-FL-TIAM1(siRNA resistant)	This study	N/A
DLD1-NLS-TIAM1(siRNA resistant)	This study	N/A
SW620-FL-TIAM1(siRNA resistant)	This study	N/A
SW620-NLS-TIAM1(siRNA resistant)	This study	N/A

**Experimental Models**: **Organisms**/**Strains**		

Mouse: *Apc*^*580S*^	Sansom et al., 2007	N/A
Mouse: *Vil-Cre-ER*	el Marjou et al., 2004	N/A
Mouse: *Tiam1*^*-*/*-*^	Malliri et al., 2002	N/A
Casper strain (*roy*^*-*/*-*^, *nacre*^*-*/*-*^) zebrafish	Gift from Richard White	White et al., 2008

**Oligonucleotides**		

See Table S4		

**Recombinant DNA**		

pSpCas9(BB)-2A-GFP	Ran et al., 2013	Addgene Plasmid #48138
pSpCas9(BB)-2A-GFP-TIAM1-KO	This study	N/A
pRetroX-Tight-Pur	Clontech	Cat# PT3968-5
pRetroX-Tight-Pur-FL-TIAM1	This study	N/A
pRetroX-Tight-Pur-NLS-TIAM1	This study	N/A
pRetroX-Tight-Pur-FL-TIAM1(siRNA resistant)	This study	N/A
pRetroX-Tight-Pur-NLS-TIAM1(siRNA resistant)	This study	N/A
pcDNA3-NLS-TIAM1	This study	N/A
pEGFP-N3 FL-TIAM1	This study	N/A
pEGFP-N3 FL-TIAM1-ΔNLS2	This study	N/A
pEGFP-N3 TIAM1-m1	This study	N/A
pEGFP-N3 TIAM1-m2	This study	N/A
pEGFP-N3 TIAM1-m3	This study	N/A
pGL3-8xTEAD Firefly vector	Dr Georges Lacaud lab	N/A
pGL3-Control Firefly vector	Dr Georges Lacaud lab	N/A
pGL4 Renilla vector	Dr Georges Lacaud lab	N/A

**Software and Algorithms**		

Universal ProbeLibrary Assay Design Center	Roche	https://lifescience.roche.com/en_gb/brands/universal-probe-library.html
Human reference genome NCBI build 37, GRCh37	Genome Reference Consortium	http://www.ncbi.nlm.nih.gov/projects/genome/assembly/grc/human/
Bowtie2 (version 2.2.1)	Langmead and Salzberg, 2012	http://bowtie-bio.sourceforge.net/bowtie2/index.shtml
Tophat2 (version 2.0.12)	Kim et al., 2013	http://ccb.jhu.edu/software/tophat.
Bioconductor package Rsubread (version 1.13.13)	Liao et al., 2014	https://bioconductor.org/packages/release/bioc/html/Rsubread.html
Bioconductor package edgeR (version 3.8.5)	Robinson et al., 2010	https://bioconductor.org/packages/release/bioc/html/edgeR.html
MACS2 (version 2.1.0.20150420)	Zhang et al., 2008	https://pypi.python.org/pypi/MACS2/2.1.0.20150420
GenomicRanges	Ramirez et al., 2014	http://bioconductor.org/packages/release/bioc/html/GenomicRanges.html
Deeptools	Lawrence et al., 2013	http://deeptools.ie-freiburg.mpg.de
GraphPad Prism version 6.0	GraphPad Software Inc	http://www.graphpad.com/scientific-software/prism/
IBM SPSS statistics 23	IBM	http://www-01.ibm.com/support/docview.wss?uid=swg24038592
Volocity Software version 6.0.1	Perkin Elmer, Cambridge, UK	http://ftparmy.com/248712-volocity.html

### Contact for Reagent and Resource Sharing

Further information and requests for resources and reagents should be directed to and will be fulfilled by the Lead Contact Angeliki Malliri (Angeliki.Malliri@cruk.manchester.ac.uk)

### Experimental Model and Subject Details

#### Mouse Experiments

All experimental procedures were performed under National Home Office guidelines and were approved by the ethical review body of Glasgow University. The *Vil-Cre-ER* experiments were performed on a mixed background (50% C57Bl6J, 50% S129). The alleles used for this study were as follows: *Apc*^*580S*^ (Sansom et al., 2007), *Vil-Cre-ER* (el Marjou et al., 2004) and *Tiam1*^-/-^ (Malliri et al., 2002). Recombination by *Vil-Cre-ER* was achieved by intraperitoneal (i.p) injection of 80 mg/kg tamoxifen per day for 2 days. Analysis of *Vil-Cre-ER* induced mice was carried out at day 4 post induction, where the intestine was placed in 10% normal buffered formalin for 16-18 hr at 4°C. Immunohistochemical analysis was performed using standard techniques. Primary antibodies and concentrations were used as follows: CTGF (1:200; Abcam, 6992) and CYR61 (1:100; Abcam, 24448).

#### Zebrafish Xenograft Assay

Adult zebrafish (*Danio rerio*) were maintained at The University of Manchester Biological Services Unit. Experiments were performed according to National Home Office regulations under the Animals (Scientific Procedures) Act 1986 and were approved by the ethical review body of The University of Manchester. Casper strain (*roy*^*-*/*-*^, *nacre*^*-*/*-*^) zebrafish were used to generate embryos completely lacking pigment which can otherwise obscure imaging. SW480 cell lines were resuspended at 1.4 x 10^7^ cells/ml on ice with PBS (w/o CaCl_2_ and MgCl_2_) supplemented with 0.5% polyvinylpyrrolidinone K60 solution (PVP, Sigma). 48 hr post fertilization (hpf) embryos were anaesthetised with 0.1 mg/ml MS-222 (Sigma) and approximately 500 cells were injected into the pericardial cavity, using a micropipette and pump (World Precision Instruments). Engrafted embryos were sorted to remove falsely injected embryos and as needed, treated with 1 μg/ml doxycycline (Fisher Scientific) and maintained at 34°C for a further 4 days. At 4 days post injection (dpi) engrafted zebrafish embryos were anaesthetised in 0.1 mg/ml MS-222, mounted in 1.5% low melting agarose (LMP, Promega) and xenografts imaged using a Leica TCS SP5 AOBS upright confocal (Leica Microsystems) using a 20x 0.50 Plan Fluotar dipping objective and 1.0x confocal zoom. Z stacks from the top to the bottom of the tumor were captured and processed using Volocity Software version 6.0.1 (Perkin Elmer) with the Volocity Visualization module and standard parameters. All experiments consist of three independent repeats. Relative Invasion Index is defined as the number of cells invaded outside the pericardial cavity at 4 dpi normalized to the average number of invading cells in the control group.

#### TMA Immunohistochemical Analysis

Immunohistochemistry for TIAM1 (1:700; R&D Systems, AF5038) was performed with a standard avidin-biotin complex method (ABC) on a colorectal cancer tissue microarray (TMA) containing 650 primary colorectal cancers all of which were histologically adenocarcinomas. TMA samples were obtained from the Grampian Biorepository with ethical approval from the scientific access group of the Grampian Biorepository (tissue request number TR72). The design and construction of the TMA has previously been described (Brown et al., 2014). Clinico-pathological details of the patients and the colorectal cancers included in the tissue microarray are summarized in Table S1. None of the patients had received either pre-operative chemotherapy or radiotherapy. Following completion of the TIAM1 immunohistochemistry the TMA slides were examined by an expert gastro-intestinal pathologist (GIM) by light microscopy using an Olympus BX 51 microscope (Olympus) equipped with an Olympus C4040 digital camera (Olympus). The intensity of immunostaining was quantified using a semi-quantitative scoring method as previously described (Brown et al., 2014). The intensity of immunostaining (negative, weak, moderate, strong) and its subcellular localization (cytoplasmic, nuclear, membranous) was assessed. Statistical analysis including survival analysis was performed using IBM SPSS statistics 23.

#### Cell Lines

DLD1 cells were cultured in RPMI supplemented with 10% fetal bovine serum, Penicillin and Streptomycin. The rest of the cells lines were cultured in Dulbecco’s Modified Eagle Medium supplemented with 10% fetal bovine serum, Penicillin and Streptomycin. Cell lines were maintained in a 5% CO_2_ incubator at 37°C. Cell lines were routinely tested to exclude *Mycoplasma* contamination.

### Method Details

#### Quantitative PCR (qPCR)

1 μg of RNA was reverse transcribed to cDNA using the Omniscript RT Kit (Qiagen) according to the manufacturer’s instructions. qPCR was performed in triplicate in a 10 μl reaction mixture containing 5 μl of 2× TaqMan® master mix, 0.5 μM of each of the primers and 10 ng cDNA. The reaction mixture without a template was run as a control. *ACTB* expression levels were used to normalize for differences in RNA input. Primers for *ACTB*, *APC*, *CTGF*, *CYR61* and *TIAM1* were designed online using the Universal ProbeLibrary Assay Design Center and listed in Table S4. For the rest of the genes predesigned TaqMan™ Gene Expression Assays (ThermoFisher Scientific) were used (*AMOTL2* #Hs01048101_m1, *ANKRD1* #Hs00173317_m1, *AXL* #Hs01064444_m1 and *WWTR1* (*TAZ*) #Hs00210007_m1).

#### RNA-Sequencing

Paired-end sequence (75 bp) were aligned to the human reference genome GRCh37 (Ensembl v75) using Tophat2 (version 2.0.12) (Kim et al., 2013) with the additional settings “--library-type fr-firststrand --no-novel-junc”. Mapped reads of the same samples across different lanes were merged into one BAM file. The expression levels of 57,773 annotated features were determined by using the featureCounts (Liao et al., 2014) function from the Bioconductor package Rsubread (version 1.13.13). The Bioconductor package edgeR (Robinson et al., 2010) (version 3.8.5) was used to identify genes that showed statistically significant variation in expression levels in the following siRNA induction: siTIAM1 versus siNT or siYAP/TAZ versus siNT. The data was filtered to include only genes with at least 1 count-per-million reads and differential expression analysis was performed using the function exactTest in edgeR (Robinson et al., 2010) (> 1.5 fold change, false discovery rate < 0.05).

#### Construction of Expression Vectors

A series of mutant pEGFP-N3 TIAM1 constructs were generated by PCR mutagenesis using pFU ultra II and QuikChange II XL Site-Directed Mutagenesis Kit (Agilent) for amino acid deletions or substitutions according to the manufacturer’s instructions. The primers used in the PCR reactions are listed in Table S4. For the NLS-TIAM1 construct, PCR was used to replace the start codon of TIAM1-HA in pcDNA3 with 5’ NLS (ATGGGTCCTCCAAAAAAGAAGAGAAAGGTA) and then NLS-TIAM1 from pcDNA3 was cloned into pRETRO X-Tight (Clontech) using appropriate restriction enzyme digests.

#### Generation of TIAM1 Knock-Out Cell Lines

A TIAM1 sgRNA sequence (5’-GCTCATCGCTGTGCGCCGAGC-3) was cloned into the pSpCas9(BB)-2A-GFP (PX458) vector (Addgene, #48138) and transfected into SW480 cells using Lipofectamine 2000 (Invitrogen). 48 hr later, single GFP positive cells were sorted and plated into 96-well plates. Surviving clones were screened for expression levels of TIAM1 by immunoblotting. Disruption of TIAM1 locus was confirmed by Sanger sequencing.

#### Generation of DLD1, SW620 and SW480 Cells Inducibly Overexpressing FL-TIAM1 or NLS-TIAM1

Phoenix cells were transfected with either pRETRO X-Tight-FL-TIAM1 or pRETRO X-Tight-NLS-TIAM1 constructs, using TransIT-LT1 (Mirus) according to the manufacturer’s instructions. Retroviral transduction was performed as previously described (Woodcock et al., 2009) and transfections were followed by appropriate antibiotic selection.

#### Transient siRNA Silencing

Transient silencing of TIAM1, TAZ, YAP or APC was achieved by transfection of siRNA oligos using Lipofectamine RNAiMAX (Invitrogen) following the manufacturer’s instructions. Cells were transfected 24 hr after seeding, re-transfected on day 4 and processed and analyzed 6 days after the first transfection by when cells had reached full confluency. Two different siRNA sequences were used for YAP1 (Ambion, #s20367 and #s20366) and TAZ (Ambion, #s24789 and #s24787) and a single siRNA for APC (Ambion, #s1433). For TIAM1, two siRNA sequences were used (#1 5’-GAGGUUGCAGAUCUGAGCA-3’, #2 5’-AGAGCGCACCUACGUGAAA-3’) and synthesized by Eurofins MWG. In all of the reported assays, a negative control siRNA was used (Ambion, Negative Control #1).

#### Luciferase Assays

Firefly luciferase reporters (100 ng) were transfected together with Renilla Luciferase reporters (100 ng) to normalize the transfection efficiency. For luciferase assays in siRNA-depleted cells, cells were first transfected with the indicated siRNAs and, after 24 hr, with plasmid DNA. Each sample was transfected in duplicate, and each experiment was repeated at least three times independently. Luciferase was measured 48 hr post-transfection of HEK293.

#### Nuclear Fractionation

For nuclear fractionation cells were incubated in Hypotonic Buffer (20 mM Tris-HCl pH 7.4, 10 mM NaCl, 3 mM MgCl_2_) for 15 min on ice and then 10% NP-40 was added to make a 0.5% buffer. After a 10 min centrifugation at 800 g, the cytoplasmic fraction (the supernatant) was collected. The nuclear pellet was resuspended in Cell Extraction buffer (ThermoFisher Scientific, FNN0011) and incubated on ice for 30 min. Finally, the nuclear fraction was collected after a 30 min centrifugation at 14,000 g to remove the insoluble material. All buffers contained a protease inhibitor cocktail (Sigma) and phosphatase inhibitor cocktails 2 and 3 (Sigma).

#### Immunoprecipitation

Protein G beads were pre-blocked with an equal volume of 5% BSA/PBS to make a 50% Protein G slurry. Cells were lysed in immunoprecipitation lysis buffer [50 mM Tris-HCl pH 7.5, 150 mM NaCl, 1% (v/v) Triton-X-100, 10% (v/v) glycerol, 2 mM EDTA, 25 mM NaF and 2 mM NaH_2_PO_4_] containing protease and phosphatase inhibitor cocktails (Sigma). Lysates were pre-cleared using IgG-conjugated Protein G beads, incubated with the specific antibodies for 2 hr at 4°C and then incubated with beads for 1 hr at 4°C with gentle mixing. Beads were then washed 5 times with lysis buffer and eluted with 20 μl of 2x SDS sample buffer. Western blotting was then performed. Primary antibodies used for IP were as follows: TIAM1 (2 μg/ml; R&D Systems, AF5038) and βTrCP (D13F10; 1:100; Cell Signaling Technology, 4394).

#### Western Blotting

Protein extracts were resolved by SDS-PAGE and transferred to nylon membranes (ImmobilonP) using standard techniques. Primary antibodies used for Western blotting were as follows: TIAM1 (1:1000; Bethyl, A300-099A), TIAM1 (1:1000; R&D Systems, AF5038), TAZ (1:200; BD Biosciences, 560235), TAZ (1:500; Sigma-Aldrich, HPA0074), YAP (D8H1X; 1:1000; Cell Signaling Technology, 14074), LMNB1(1:1000; Cell Signaling Technology, 12586), βTrCP (D13F10; 1:1000; Cell Signaling Technology, 4394), βTrCP (1B1D2; 1:50; Invitrogen, 37-3400), AXIN (C76H11; 1:1000; Cell Signaling Technology, 2087), β-catenin (D10A8; 1:1000; Cell Signaling Technology, 8480), TEAD4 (1:1000; Abcam, ab58310), panTEAD (D3F7L; 1:1000; Cell Signaling Technology, 13295), CTGF (L-20; 1:200; SantaCruz Biotechnology, sc14939) and α-tubulin (DM1A; 1:2000; Sigma-Aldrich, T9026).

#### In Vitro Interaction Assay

Recombinant TIAM1 (1 μg/ml) isolated from insect cell cultures as previously described (Whalley et al., 2015) was incubated with different concentrations of recombinant TAZ (Abnova H0006901-P01) for 3 hr at 4°C and then TIAM1 was immunoprecipitated using anti-TIAM1-Ab conjugated to Protein G beads for 1 hr at 4°C with gentle mixing. Beads were then washed 5 times with lysis buffer and eluted with 20 μl of 2x SDS sample buffer. Western blotting was then performed.

#### Immunofluorescence Microscopy

Cells were grown on glass coverslips and fixed in 3.7% formaldehyde. Post-fixation, cells were washed with PBS, permeabilized for 5 min in 0.5% TritonX-100/PBS and blocked with 5% BSA in 0.1% Triton/PBS for 1 hr before TIAM1 antibody (1:100; R&D Systems, AF5038) was added.

#### Transwell Assay

Migration assays were carried out in Boyden chambers using filters (8-μm pore size, Costar). Assay medium (DMEM AQ medium supplemented with 20% FBS) was added to the lower compartment, and SW480 and SW620 colon cancer cells, treated as indicated, were added on top of the insert. After incubation for 24 hr at 37°C, filters were fixed. Non-migrated cells were scraped off the upper side of the filters and the filters were stained with crystal violet. Images were taken under a phase-contrast microscope. The number of migrated cells was quantified after Crystal Violet elution with 30% (v/v) acetic acid in distilled H_2_O and 100 μl transferred to a 96-well plate. Absorbance was read at 595 nM.

#### Proliferation Assay

Cells were transfected 24 hr after seeding and re-transfected on day 3 and 5. Where indicated, treatment with 10 ng/ml Doxycycline was performed on day 3 and 5. 6 days after the first transfection cell number was estimated by the crystal violet assay. Briefly, adherent cells were fixed with methanol and stained with 0.5% crystal violet in 20% methanol for 20 min. After gentle rinsing with water, the retained dye was extracted with 30% (v/v) acetic acid and the absorbance measured at 595 nM.

#### Duolink PLA®

This technique was performed using the Duolink II Red Starter Kit (Sigma). A video summarizing the steps of this technique can be found online (www.olink.com/products-services/duolink/how-useduolink). Briefly, cells were prepared as above for immunofluorescence before being incubated overnight at 4°C with primary antibodies against TIAM1 [(1:100; Bethyl, A300-099A) or (1:100; R&D Systems, AF5038)], TAZ [(1:100; BD Biosciences, 560235) or (1:100; Sigma-Aldrich, HPA0074)], YAP (D8H1X; 1:100; Cell Signaling Technology, 14074) and TEAD4 (1:100; Abcam, ab58310). Slides were then incubated for 1 hr at 37°C with a mix of the MINUS and PLUS PLA probes. Hybridized probes were ligated using the Ligation-Ligase solution for 30 min at 37°C and then amplified utilizing the Amplification-Polymerase solution for 100 min at 37°C. Slides were finally mounted using Duolink II Mounting Medium with DAPI.

#### Chromatin Immunoprecipitation and Next Generation Sequencing (ChIP-seq)

Chromatin immunoprecipitation was performed using the SimpleChIP® Enzymatic Chromatin IP Kit (Magnetic Beads) (Cell Signaling Technology, 9003) following the instructions of the manufacturer. 10 μg of chromatin was incubated overnight with TAZ antibody (2 μg/ml; Sigma-Aldrich, HPA0074). The MicroPlex Library Preparation Kit v2 (Diagenode) was used to prepare indexed libraries from 1 ng of ChIP DNA. The resulting libraries were then size-selected (selecting a 200-800 bp range) by adding 0.55x volume of AMPure XP beads (Beckman Coulter). To the supernatant, 0.3x the original sample volume of AMPure XP beads were added. The supernatant was discarded and the beads washed with 70% ethanol before drying and eluting the size-selected library. Library quality was checked using the Agilent Bioanalyzer. Libraries were quantified by qPCR using the KAPA Library Quantification Kit for Illumina (Kapa Biosystems Inc.). 1.7 pM pooled libraries were loaded onto the NextSeq 500 and 2x 75 bp sequencing was carried out using a NextSeq 500/550 High Output v2 kit (Illumina Inc.).

#### ChIP-seq Analysis

Paired-end sequence (75 bp) was aligned to the human reference genome GRCh37 (Ensembl v75) using bowtie2 (Langmead and Salzberg, 2012) (version 2.2.1) with default settings. Mapped reads of the same samples across different lanes were merged into one BAM file. Duplicated reads were removed with Picard tools. Peak calling was performed using MACS2 (Zhang et al., 2008) (version 2.1.0.20150420) with the call peak function and the parameter “-p 0.01”. Peaks were only kept if they had > 15 reads. We proceeded to identify all overlapping peaks whereby one or more peaks from the WT and KO samples overlapped and extend the boundaries of these regions to the maximum peak width. Peak overlap was identified using GenomicRanges (Ramirez et al., 2014) packages in R. We then calculated the RPKM (reads per kilo base per million reads) in the overlapping region and calculated the fold change. Heatmap visualization of overlapping peaks was performed by deeptools (Lawrence et al., 2013).

### Quantification and Statistical Analysis

#### Statistics

The specific statistical tests used are indicated in the figure legends alongside the p values and were carried out using GraphPad Prism version 6.0. Statistical analysis for the TMA immunohistochemical and survival analysis was performed using IBM SPSS statistics 23.

### Data and Software Availability

#### Data Resources

The RNA-seq and ChIP-seq data reported in this paper has been deposited to NCBI GEO (http://www.ncbi.nlm.nih.gov/geo) under the accession number GSE90492.

## Author Contributions

Z.D. performed the majority of experiments, data analysis, and manuscript preparation. G.W. was responsible for cloning multiple constructs, generating SW480 CRISPR-mediated TIAM1 knockout cell lines as well as cells inducibly expressing FL-TIAM1 or NLS-TIAM1, and performed several western analyses. M.Z.H.F. performed the bioinformatics analyses of RNA-seq and ChIP-seq under the supervision of V.K. and G.L. M.D. performed the zebrafish xenograft assays under the supervision of A.F.L.H., who also made intellectual contributions. K.P. performed immunohistochemistry for CTGF and CYR61 on mouse intestines under the supervision of O.J.S. J.M. generated the TIAM1 NLS mutants. R.M. performed peptide array interaction assays confirming the TAZ/TIAM1 direct interaction under the supervision of G.S.B. G.A. performed the immunohistochemistry on the human colon TMA. G.I.M. is the pathologist who scored the TMA. A.M. was the grant holder and principal investigator who supervised the study, edited the manuscript, and made intellectual contributions throughout.

## Supporting Citations

The following reference appears in the Supplemental Information: Shalem et al., 2014.

## Supplemental Information

Document S1. Figures S1–S7 and Tables S1 and S4

Table S2. TIAM1 Differentially Expressed Genes, Related to Figure 3

Table S3. TAZ/YAP Differentially Expressed Genes, Related to Figure 3

Document S2. Article plus Supplemental Information
