# Supplementary material for: TIAM1 Antagonizes TAZ/YAP Both in the Destruction Complex in the Cytoplasm and in the Nucleus to Inhibit Invasion of Intestinal Epithelial Cells
Source: Cancer Cell. 2017 May 8;31(5):621–634.e6. doi: 10.1016/j.ccell.2017.03.007 (PMC5425402; doi:10.1016/j.ccell.2017.03.007)
Supplement: Document S1. Figures S1–S7 and Tables S1 and S4 [file mmc1.pdf]

Cancer Cell, Volume *31*

Supplemental Information

TIAM1 Antagonizes TAZ/YAP Both in the Destruction Complex in the Cytoplasm and in the Nucleus to Inhibit Invasion of Intestinal Epithelial Cells

Zoi Diamantopoulou, Gavin White, Muhammad Z.H. Fadlullah, Marcel Dreger, Karen Pickering, Joe Maltas, Garry Ashton, Ruth MacLeod, George S. Baillie, Valerie Kouskoff, Georges Lacaud, Graeme I. Murray, Owen J. Sansom, Adam F.L. Hurlstone, and Angeliki Malliri

A

		Dukes stage			Total
		A	B	C	
Nuclear TIAM1	0	16	49	89	154
	1	59	147	143	349
	2	39	39	33	111
	3	5	1	0	6
Total		119	236	265	620

Chi-Square Tests

	Value	df	Asymptotic Significance (2-sided)
Pearson Chi-Square	54.165[a]	6	0.000

B

		Dukes stage			Total
		A	B	C	
Cytoplasmic TIAM1	0	78	184	223	485
	1	40	48	38	126
	2	1	4	4	9
Total		119	236	265	620

Chi-Square Tests

	Value	df	Asymptotic Significance (2-sided)
Pearson Chi-Square	19.057[a]	4	0.001

C

Case Processing Summary

Nuclear TIAM1	Total N	N of Events	Censored	
			N	Percent
Low	503	251	252	50.1%
High	117	42	75	64.1%
Overall	620	293	327	52.7%

Means and Medians for Survival Time

Nuclear TIAM1	Mean[a]				Median			
	Estimate	Std. Error	95% Confidence Interval		Estimate	Std. Error	95% Confidence Interval	
			Lower Bound	Upper Bound			Lower Bound	Upper Bound
Low	113.125	4.494	104.316	121.934	92.000	9.107	74.151	109.849
High	114.593	7.052	100.772	128.415	125.000	.	.	.
Overall	116.907	4.157	108.760	125.055	107.000	8.893	89.569	124.431

a. Estimation is limited to the largest survival time if it is censored.

Overall Comparisons

	Chi-Square	df	Sig.
Log Rank (Mantel-Cox)	5.010	1	0.025

D

Case Processing Summary

Cytoplasmic TIAM1	Total N	N of Events	Censored	
			N	Percent
0	485	235	250	51.5%
1	126	53	73	57.9%
2	9	5	4	44.4%
Overall	620	293	327	52.7%

Means and Medians for Survival Time

Cytoplasmic TIAM1	Mean[a]				Median			
	Estimate	Std. Error	95% Confidence Interval		Estimate	Std. Error	95% Confidence Interval	
			Lower Bound	Upper Bound			Lower Bound	Upper Bound
0	111.192	4.495	102.381	120.003	95.000	11.497	72.465	117.535
1	129.675	8.840	112.347	147.002	109.000	12.797	83.918	134.082
2	93.778	24.777	45.214	142.342	55.000	38.759	0.000	130.967
Overall	116.907	4.157	108.760	125.055	107.000	8.893	89.569	124.431

Pairwise Comparisons

	Cytoplasmic TIAM1	0		1		2	
		Chi-Square	Sig.	Chi-Square	Sig.	Chi-Square	Sig.
Log Rank (Mantel-Cox)	0			2.617	0.106	0.000	0.994
	1	2.617	0.106			0.517	0.472
	2	0.000	0.994	0.517	0.472		

Figure S1, related to Figure 1. Abundance of nuclear TIAM1 impacts on CRC progression.
(A, B) Pearson Chi-Square test to analyze the correlation between the nuclear intensity of TIAM1 (A) and the cytoplasmic intensity of TIAM1 (B) and different Dukes stages of CRC patients.
(C, D) Mantel-Cox analysis to compare the overall-survival distribution of patients with high and low intensity of nuclear (C) or cytoplasmic (D) TIAM1 staining.

Table S1, related to Figure 1. Clinico-pathological characteristics of patients and their tumors included in the colorectal cancer tissue microarray.

	Number of patients	Percentage	Relationship with overall survival
Sex			
Male	340	52.3	$\chi^2 = 0.027$, p = 0.870
Female	310	47.7	
Age			
< 70	305	46.9	$\chi^2 = 29.213$, **p < 0.001**
≥ 70	345	53.1	
Bowel cancer screening detected			
Yes	52	8	$\chi^2 = 16.381$, **p < 0.001**
No	598	92	
Tumor site			
Proximal colon	261	40.2	Proximal v distal, $\chi^2 = 8.418$, **p = 0.004**
Distal colon	245	37.7	Distal v rectal, $\chi^2 = 0.906$, p = 0.341
Rectum	144	22.2	Colon v rectum, $\chi^2 = 0.098$, p = 0.754
Tumor differentiation			
Well/moderate	600	92.3	$\chi^2 = 0.976$, p = 0.323
Poor	50	7.7	
Extra mural venous invasion			
Present	140	21.5	$\chi^2 = 100.946$, **p < 0.001**
Absent	510	78.5	
Mismatch repair protein status			
Defective	96	15.2	$\chi^2 = 2.848$, p = 0.091
Proficient	536	84.8	
Tumor (pT) stage			
pT1	30	4.6	T1 v T2, $\chi^2 = 0.382$, p = 0.536
pT2	114	17.5	T2 v T3, $\chi^2 = 24.739$, **p < 0.001**
pT3	411	63.2	T3 v T4, $\chi^2 = 30.159$, **p < 0.001**
pT4	95	14.6	
Lymph node (pN) stage			
pN0	364	56	N0 v N1, $\chi^2 = 54.071$, **p < 0.001**
pN1	177	27.2	N1 v N2, $\chi^2 = 17.636$, **p < 0.001**
pN2	109	16.8	
Dukes stage			
A	120	18.5	A v B, $\chi^2 = 5.059$, **p = 0.025**
B	244	37.5	B v C, $\chi^2 = 65.510$, **p < 0.001**
C	286	44	

Significant values are highlighted in bold.

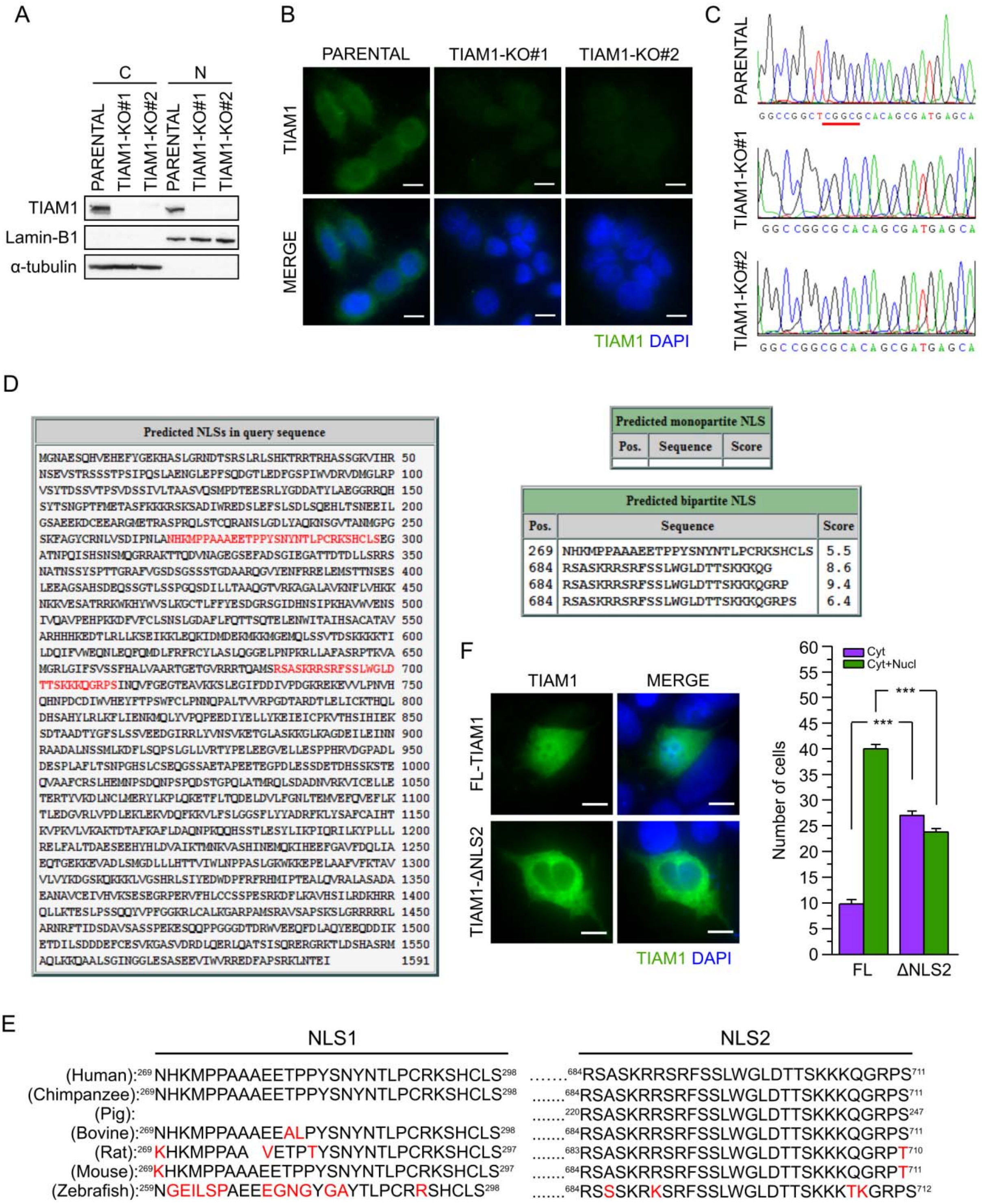

Figure S2, related to Figure 2. TIAM1 nucleocytoplasmic shuttling is regulated by a bipartite NLS.
(A) Western blot analyses of cytoplasmic (C) and nuclear (N) fractions from SW480 cells or the CRISPR-mediated TIAM1 knock-out clones (TIAM1-KO#1, TIAM1-KO#2). α-tubulin and Lamin-B1 were used as cytoplasmic and nuclear markers, respectively. Figure representative of three independent experiments.
(B) Representative confocal images from three independent experiments of endogenous TIAM1 in SW480 cells or the CRISPR-mediated TIAM1 knock-out clones. Scale bars, 10 µm.
(C) Sequencing results for the TIAM1 locus from genomic DNA isolated from SW480 cells or the CRISPR-mediated TIAM1 knock-out clones.
(D) In silico analysis of TIAM1 sequence for the prediction of putative NLSs.

(E) Alignment of TIAM1 protein sequence from different species to determine the conservation and homology of potential TIAM1 NLSs among various species.

(F) Representative confocal images of DLD1 cells transiently transfected with GFP-tagged FL-TIAM1 and TIAM1-ΔNLS2 constructs. Graph shows quantitation from three independent experiments ($n = 50$ cells per experiment) and are presented as mean ± SEM (unpaired t-test, ***$p < 0.001$). Scale bars, 10 μm.

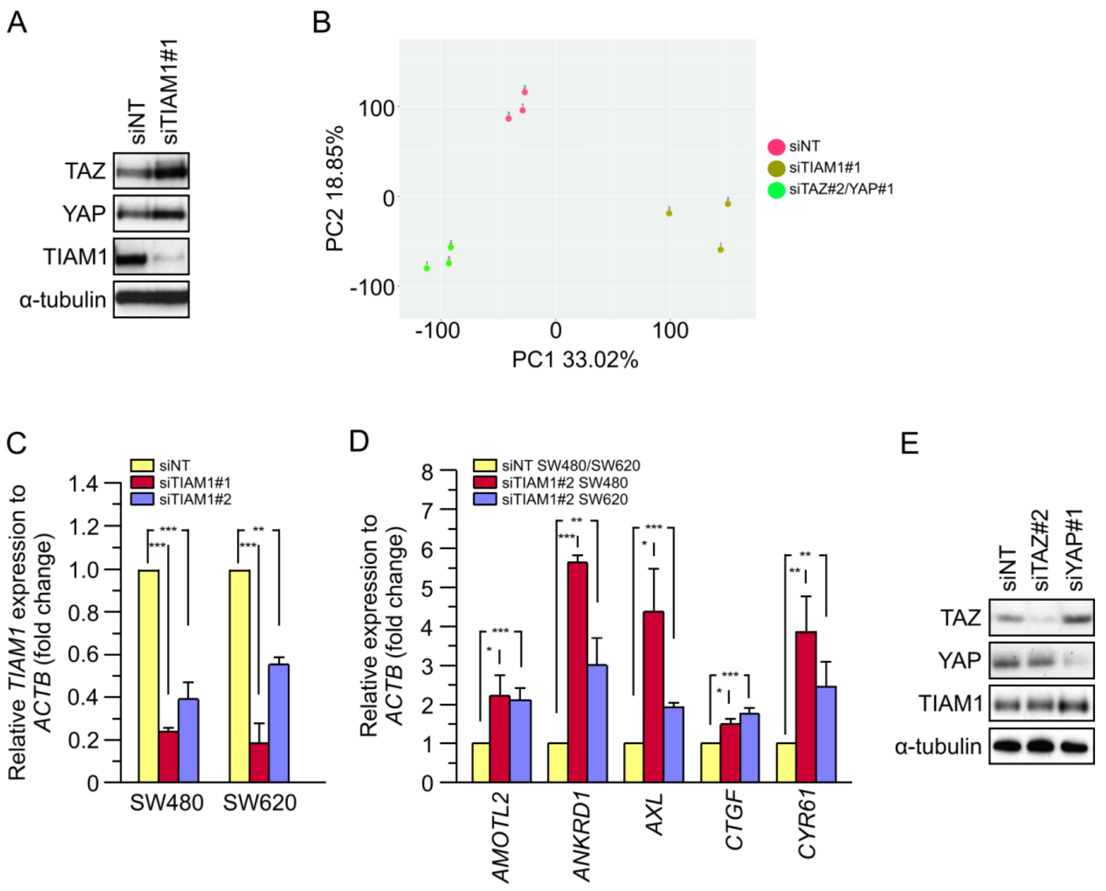

Figure S3, related to Figure 3. TIAM1 suppresses a TAZ/YAP transcriptional program.
(A) Western blot analyses of endogenous proteins in SW620 cells transfected with control siRNA (siNT) or TIAM1#1 siRNA as indicated.
(B) Principal component analysis of RNA-seq expression data from all biological replicates of siNT, siTIAM1#1 and siTAZ/YAP transfected SW620 cells (n = 3).
(C) qPCR for *TIAM1* normalized to *ACTB* expression in SW480 and SW620 cells transfected with TIAM1#1 or TIAM1#2 siRNA.
(D) qPCR for TAZ/YAP target genes *AMOTL2*, *ANKRD1*, *AXL*, *CTGF* and *CYR61* normalized to *ACTB* expression in SW620 and SW480 cells transfected with TIAM1#2 siRNA.
(E) Western blot analyses of endogenous proteins in SW620 cells transfected with YAP or TAZ siRNAs or siNT as indicated.
Data are relative to negative control siRNA and are presented as mean ± SEM (with siNT set as 1) (unpaired t-test, *p < 0.05, **p < 0.01, ***p < 0.001).

Table S2, related to Figure 3. TIAM1 differentially expressed genes. Provided as an Excel file.

Table S3, related to Figure 3. TAZ/YAP differentially expressed genes. Provided as an Excel file.

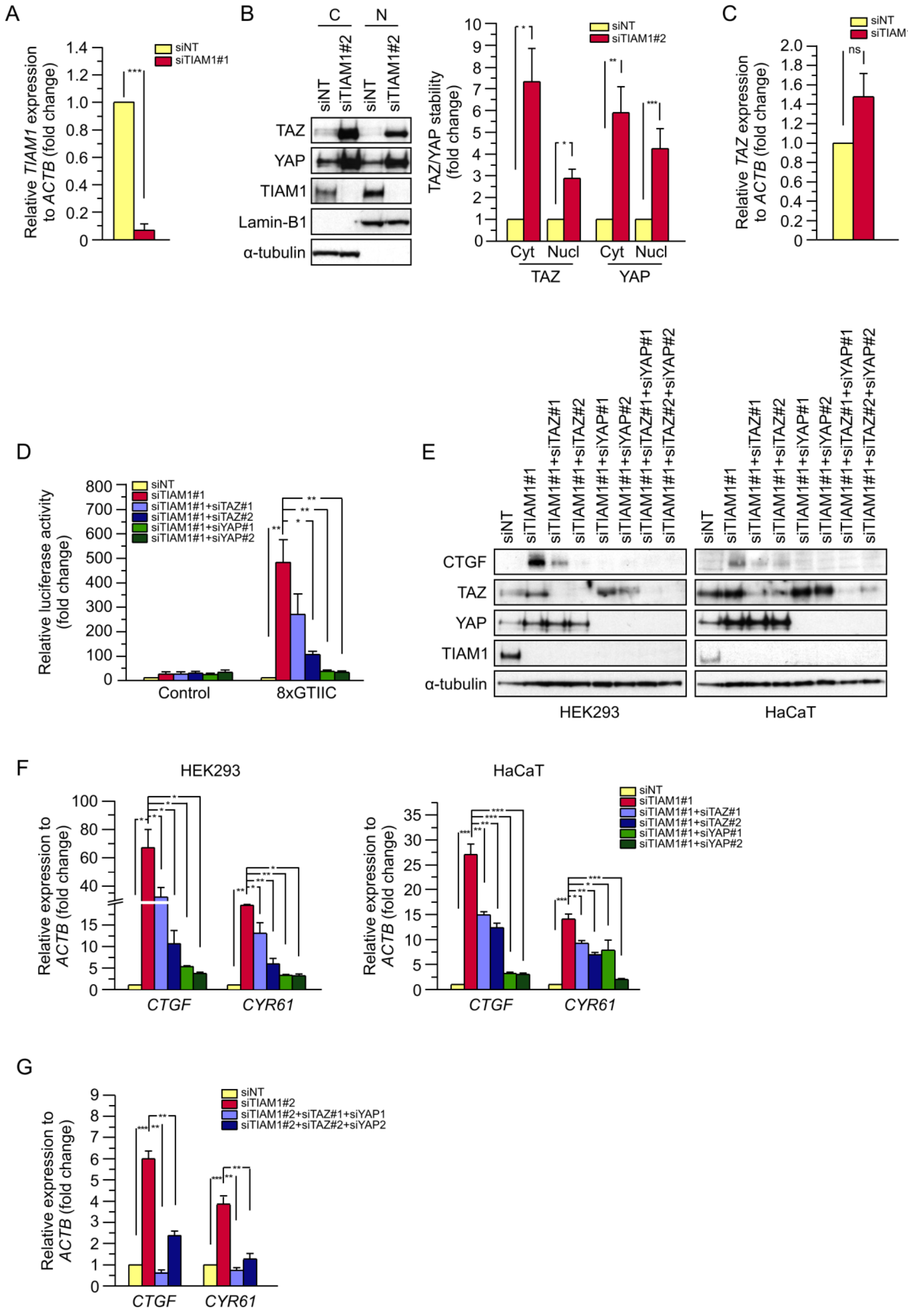
A
siNT
siTIAM1#1
Relative TIAM1 expression to ACTB (fold change)
***
B
C
N
siNT
siTIAM1#2
siNT
siTIAM1#2
TAZ
YAP
TIAM1
Lamin-B1
α-tubulin
TAZ/YAP stability (fold change)
siNT
siTIAM1#2
Cyt
Nucl
Cyt
Nucl
TAZ
YAP
C
siNT
siTIAM1#1
ns
Relative TAZ expression to ACTB (fold change)
D
Relative luciferase activity (fold change)
siNT
siTIAM1#1
siTIAM1#1+siTAZ#1
siTIAM1#1+siTAZ#2
siTIAM1#1+siYAP#1
siTIAM1#1+siYAP#2
Control
8xGTIIC
E
siNT
siTIAM1#1
siTIAM1#1+siTAZ#1
siTIAM1#1+siTAZ#2
siTIAM1#1+siYAP#1
siTIAM1#1+siYAP#2
siTIAM1#1+siTAZ#1+siYAP#1
siTIAM1#1+siTAZ#2+siYAP#2
CTGF
TAZ
YAP
TIAM1
α-tubulin
HEK293
HaCaT
F
HEK293
HaCaT
Relative expression to ACTB (fold change)
siNT
siTIAM1#1
siTIAM1#1+siTAZ#1
siTIAM1#1+siTAZ#2
siTIAM1#1+siYAP#1
siTIAM1#1+siYAP#2
CTGF
CYR61
G
Relative expression to ACTB (fold change)
siNT
siTIAM1#2
siTIAM1#2+siTAZ#1+siYAP1
siTIAM1#2+siTAZ#2+siYAP2
CTGF
CYR61

Figure S4, related to Figure 4. TIAM1 regulates TAZ stability and nuclear translocation.
(A) qPCR for *TIAM1* normalized to *ACTB* expression in confluent HEK293 cells transfected with TIAM1#1 siRNA.
(B) Western blot analyses of cytoplasmic (C) and nuclear (N) fractions from confluent HEK293 cells transfected with TIAM1#2 siRNA. Graph shows mean TAZ and YAP levels normalized to cytoplasmic (α-tubulin) or nuclear (Lamin-B1) markers and then to negative control siRNA (siNT) from three independent replicates.
(C) qPCR for *TAZ* normalized to *ACTB* expression in confluent HEK293 cells transfected with TIAM1#1 siRNA.
(D) Luciferase assay using 8xGTIIC-Lux or control reporter indicating TAZ/YAP-dependent transcriptional activity in confluent HEK293 cells transfected with TIAM1#1 siRNA, either alone or with two different TAZ/YAP siRNAs as indicated. Data are normalized to a co-transfected Renilla reporter and to negative control siRNA (siNT).
(E) Western blot analyses of endogenous proteins in HEK293 or HaCaT cells transfected with TIAM1#1 siRNA, either alone or with two different YAP or TAZ siRNAs (or pairs thereof) as indicated. Figure representative of three independent experiments.
(F) qPCR for TAZ/YAP target genes *CTGF* and *CYR61* normalized to *ACTB* expression in confluent HEK293 and HaCaT cells transfected with TIAM1#1 siRNA, either alone or with two different YAP or TAZ siRNAs as indicated.
(G) qPCR for TAZ/YAP target genes *CTGF* and *CYR61* normalized to *ACTB* expression in confluent HEK293 cells transfected with TIAM1#2 siRNA, either alone or with two different YAP or TAZ siRNAs as indicated. Data are normalized to negative control siRNA (siNT) and are presented as mean ± SEM (with siNT set as 1) (unpaired t-test, *$p < 0.05$, **$p < 0.01$, ***$p < 0.001$, ns: not significant).

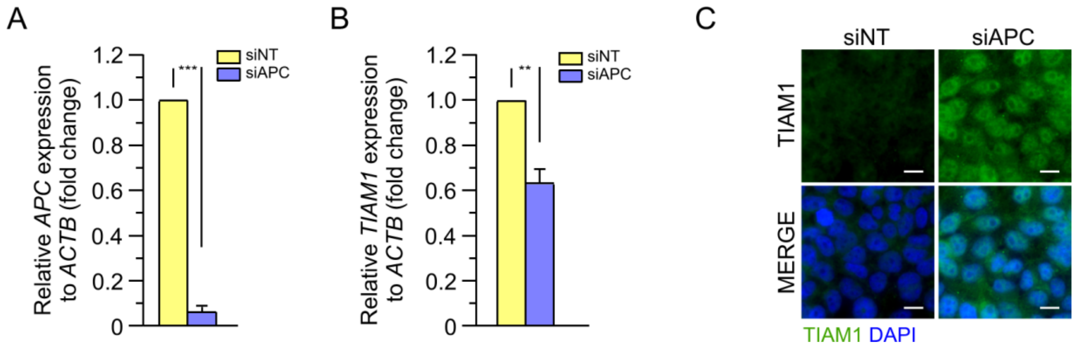

Figure S5, related to Figure 5. TIAM1 is regulated by the canonical WNT pathway.

(A, B) qPCR for *APC* (A) and *TIAM1* (B) normalized to *ACTB* expression in confluent HEK293 cells transfected with APC siRNA. Data are normalized to negative control siRNA (siNT) and are presented as mean ± SEM (with siNT set as 1) (unpaired t-test, $^{**}p < 0.01$, $^{***}p < 0.001$).

(C) Representative confocal images of TIAM1 localization in confluent HEK293 cells transfected with control (siNT) or APC siRNA. Scale bars, 10 μm.

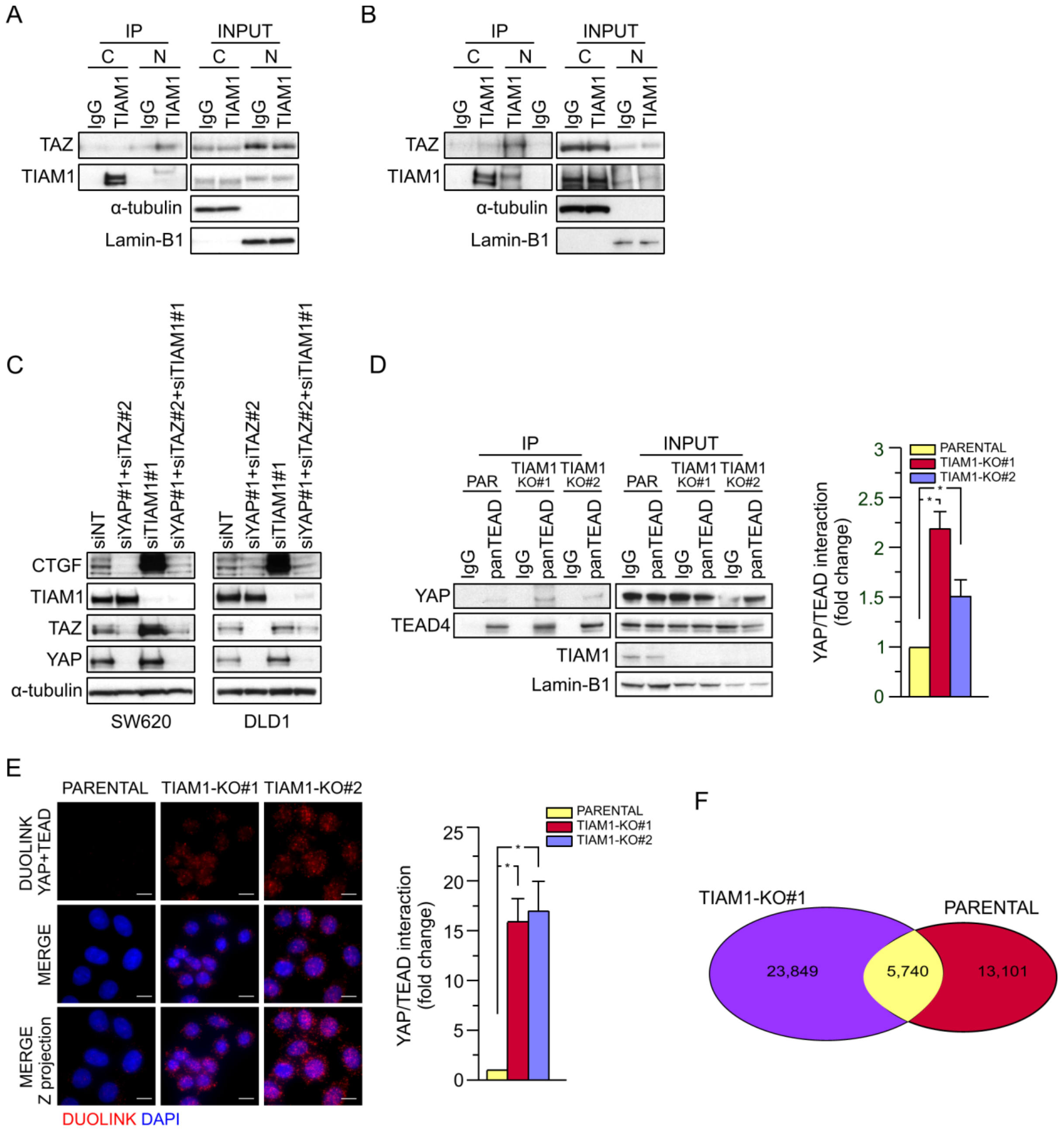

Figure S6, related to Figure 6. Nuclear TIAM1 antagonizes TAZ transcriptional activity.

(A, B) Western blot analyses of endogenous TAZ co-immunoprecipitating with endogenous TIAM1 from cytoplasmic (C) and nuclear (N) fractions of SW620 (A) or SW480 (B) cells. IgG was used as a control antibody for immunoprecipitation. α-tubulin and Lamin-B1 were used as cytoplasmic and nuclear markers, respectively. Figure representative of three independent experiments.

(C) Western blot analyses of endogenous proteins in SW620 or DLD1 cells transfected with TIAM1#1, YAP#1 and TAZ#2 siRNAs or all three siRNAs as indicated. Figure representative of three independent experiments.

(D) Western blot analyses of endogenous YAP co-immunoprecipitating with endogenous TEAD from nuclear fractions of parental SW480 cells or the CRISPR-mediated TIAM1 knock-out clones. IgG was used as a control antibody for immunoprecipitation. Graph shows mean levels of immunoprecipitated YAP normalized to levels of immunoprecipitated TEAD from three independent replicates.

(E) Representative confocal images of YAP/TEAD interaction in parental SW480 cells or the CRISPR-mediated TIAM1 knock-out clones detected by DUOLINK assay. Graph shows quantitation from three independent experiments Scale bars, 10 μm.

(F) Venn diagram showing overlap of TAZ-associated genomic DNA peaks in parental and TIAM1-KO#1 SW480 cells as determined by ChIP-seq analysis.

Data shown in graphs are presented as mean ± SEM with parental set as 1 (unpaired t-test, $*p < 0.05$).

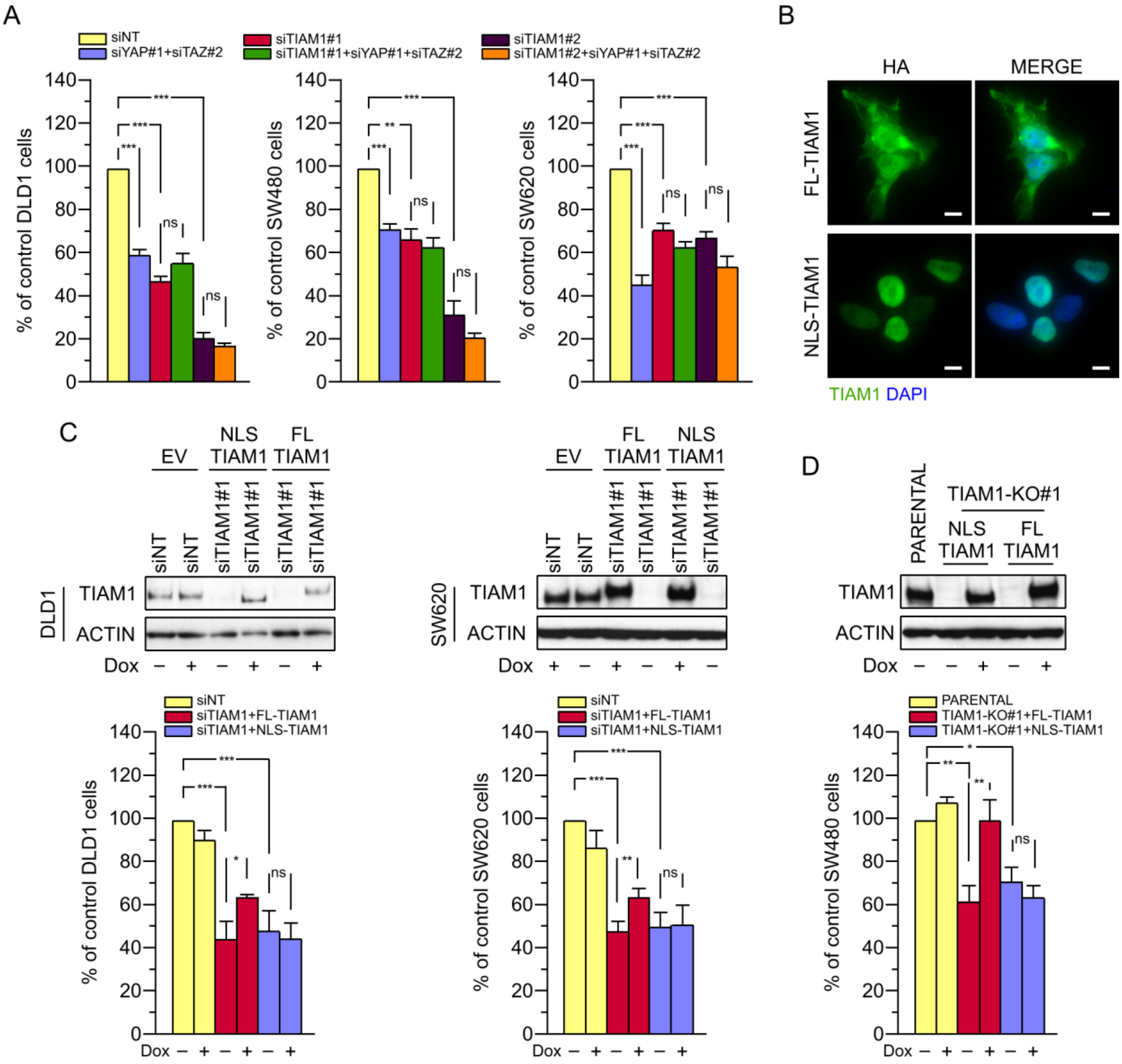

Figure S7, related to Figure 7. Effect of TIAM1 on cell proliferation.

(A) Average number of DLD1, SW480 and SW620 cells 6 days following transfection with siRNA for TIAM1 or TAZ/YAP either alone or in combination, as indicated. Results were normalized to negative control siRNA (siNT).

(B) Representative confocal images of HEK293 cells transiently transfected with HA-tagged FL-TIAM1 or NLS-TIAM1 constructs stained for the HA tag and counterstained with DAPI. Scale bars, 10 µm.

(C) Representative western blots from three independent experiments for DLD1 and SW620 cells transfected with TIAM1#1 siRNA inducibly expressing either FL-TIAM1 or NLS-TIAM1 resistant to siTIAM1#1 following addition of 10 ng/ml Doxycycline (Plus Dox). ACTIN is used as a loading control. Graphs show changes in cell number 6 days following siRNA transfection and Doxycycline addition.

(D) Representative western blots from three independent experiments of parental and SW480 CRISPR-mediated TIAM1 knock-out cells (TIAM1-KO#1 clone) inducibly expressing either FL-TIAM1 or NLS-TIAM1 following addition of 10 ng/ml Doxycycline (Plus Dox). ACTIN is used as a loading control. Graph shows changes in cell number 6 days following Doxycycline addition.

Data are presented as mean ± SEM with siNT (A, C) or parental (D) set as 100 (unpaired t-test, *$p < 0.05$, **$p < 0.01$, ***$p < 0.001$, ns: not significant).

Table S4, related to Key Resources Table. List of oligonucleotides used in this study.

**Oligonucleotides**		
qPCR Primers: *ACTB*-Fwd: 5'-ATTGGCAATGAGCGGTTC-3' and Rev: 5'-GGATGCCACAGGACTCCAT-3'	This study	N/A
qPCR Primers: *APC*-Fwd: 5'-GACCGTTTCCTCAGGTGCTA-3' and Rev: 5'-AATGGGACAGTCCTCAATTCTC-3'	This study	N/A
qPCR Primers: *CTGF*-Fwd: 5'-CCTGCAGGCTAGAGAAGCAG-3' and Rev: 5'-TGGAGATTTTGGGAGTACGG-3'	This study	N/A
qPCR Primers: *CYR61*-Fwd: 5'-CCAGTGTACAGCAGCCTGAA-3' and Rev: 5'-GGCCGGTATTTCTTCACACTC-3'	This study	N/A
qPCR Primers: *TIAM1*-Fwd: 5'-CCATGAGCAGGGCAGTGT-3' and Rev: 5'-CGGAGACGGCATCAGAAT-3'	This study	N/A
qPCR Primers: *AMOTL2*	ThermoFisher Scientific	Cat# Hs01048101_m1
qPCR Primers: *ANKRD1*	ThermoFisher Scientific	Cat# Hs00173317_m1
qPCR Primers: *AXL*	ThermoFisher Scientific	Cat# Hs01064444_m1
qPCR Primers: *WWTR1 (TAZ)*	ThermoFisher Scientific	Cat# Hs00210007_m1
Cloning primer: NLS-TIAM1: 5'-ATGGGTCCTCCAAAAAAGAAGAGAAAGGTA-3'	This study	N/A
Cloning primer: TIAM1ΔNLS2-Fwd: 5'-ACCATCAACCAGGTGTTTGGA-3' and Rev: 5'-AGGCGTACTCAGGCCATGTCC-3'	This study	N/A
Cloning primer: TIAM1-m1-Fwd: 5'-CCAGAGAGAAGAAAACCTGCTCGCTGCCGCGCTTGCAGACCTGGACATGGCC-3' and Rev: 5'-GGCCATGTCCAGGTCTGCAAGCGCGGCAGCGAGCAGGTTTTCTTCTCTCTGG-3'	This study	N/A
Cloning primer: TIAM1-m2-Fwd: 5'-GTTGATGGTTGGGCGTCCCTGCGCCGCTGCGGAGGTAGTGTCTAGGCCCCAG-3' and Rev: 5'-CTGGGGCCTAGACACTACCTCCGCAGCGGCGCAGGGACGCCCAACCATCAAC-3'	This study	N/A
sgRNA sequence: TIAM1-KO: 5'-GCTCATCGCTGTGCGCCGAGC-3	Shalem et al., 2014	N/A
siRNA sequence for *APC*	ThermoFisher Scientific	Cat# s1433
siRNA#1 sequence for *YAP1*	ThermoFisher Scientific	Cat# s20366
siRNA#2 sequence for *YAP1*	ThermoFisher Scientific	Cat# s20367
siRNA#1 sequence for *WWTR1 (TAZ)*	ThermoFisher Scientific	Cat# s24789
siRNA#2 sequence for *WWTR1 (TAZ)*	ThermoFisher Scientific	Cat# s24787
siRNA#1 sequence for *TIAM1*: 5'-GAGGUUGCAGAUCUGAGCA-3'	Vaughan et al., 2015	N/A
siRNA#2 sequence for *TIAM1*: 5'-AGAGCGCACCUACGUGAAA-3	Vaughan et al., 2015	N/A
siRNA sequence for NT	ThermoFisher Scientific	Negative Control #1
